# Outcome parameters in studies investigating dry eye disease: A systematic literature review

**DOI:** 10.1111/aos.70031

**Published:** 2025-11-20

**Authors:** Viktoria Pai, Doreen Schmidl, Liudmyla Petric, Patrick Janku, Theresa Lindner, Ulrich Graf, Jacqueline Chua, Leopold Schmetterer, Gerhard Garhöfer

**Affiliations:** ^1^ Department of Clinical Pharmacology Medical University of Vienna Vienna Austria; ^2^ Singapore Eye Research Institute Singapore National Eye Centre Singapore Singapore; ^3^ Ophthalmology and Visual Sciences Academic Clinical Program Duke‐NUS Medical School Singapore Singapore; ^4^ Center for Medical Physics and Biomedical Engineering Medical University of Vienna Vienna Austria; ^5^ School of Chemistry, Chemical Engineering and Biotechnology Nanyang Technological University Singapore Singapore; ^6^ SERI‐NTU Advanced Ocular Engineering (STANCE) Nanyang Technological University Singapore Singapore; ^7^ Fondation Ophtalmologique Adolphe De Rothschild Paris France

**Keywords:** dry eye syndromes, signs and symptoms, systematic review, treatment outcome

## Abstract

The development of effective therapeutics for dry eye disease (DED) is challenging due to its complex pathophysiology, heterogeneous patient presentation and the significant failure rate of previous clinical development programs. This underlines the importance of the selection of appropriate endpoints for clinical trials. The presented systematic review retrospectively analyzes the endpoints used in controlled clinical trials in studies of DED. Published clinical trials in the field of DED were reviewed from 2000 to 2023 and the used clinical endpoints were recorded by type and frequency of use. All studies that met the primary keyword search across various databases (PubMed, Embase, The Cochrane Library, Web of Science and Medline) were imported into Rayyan which was used to facilitate the screening process and support the collaboration between the reviewers. 93 876 studies were found of which 33 908 remained after duplicates were removed. All abstracts were screened for eligibility independently by two reviewers. 355 articles remained for full‐text review, of which 194 were included in the present systematic review. The most frequently investigated product was topical medicinal products (88 studies), followed by topical lubricants (57 studies) and nutritional supplements (22 studies). Corneal fluorescein staining (45 studies) and the ocular surface disease index (OSDI; 96 studies) were the most frequently used primary objective and subjective outcome parameters. However, a sustainable number of studies failed to show statistically significant differences between treatment and control groups, despite improvements from baseline. Our findings show that corneal fluorescein staining and OSDI are the most frequently used endpoints in clinical studies, although they frequently are not able to detect differences between the treatment and control groups. Therefore, to enhance the efficiency and reliability of DED clinical trials, a consensus on optimal outcome measures is crucial, and the exploration of novel endpoints should be prioritized. PROSPERO Registration Number: CRD42022350817.

## 1 INTRODUCTION

Dry eye disease (DED) is a common ocular disorder with a significant impact on the well‐being and quality of life of patients (Stapleton et al., 2017). Over the past two decades, great progress has been made in understanding the pathogenesis of the disease and a significant number of promising drug candidates have been identified and entered clinical development (Bron et al., 2017; Pflugfelder & de Paiva, 2017). However, the number of approved pharmacotherapies for the treatment of DED is still low (Gupta et al., 2020). The high number of failures in late stages of clinical development, particularly in phase 3, may be least partly related to the complex interaction between clinical signs and patient‐related outcomes, which complicates the design of clinical trials (Garhöfer et al., 2020; Johnson, 2009). Thus, as for many of the drugs, efficacy is observed only in either signs or symptoms—or both but in different pivotal trials (Shen Lee et al., 2022). This is, however, of importance since regulators usually require a convincing improvement of both signs and symptoms for drug approval in DED. The situation is further complicated due to ‘placebo response’, where even pharmacologically inert agents can have a measurable effect and may therefore mask the effects of the drug (Montecchi‐Palmer et al., 2023).

Thus, selecting an appropriate parameter to monitor disease progression and clinical efficacy is of particular interest (Novack et al., 2017). However, in contrast to the back of the eye, where a large body of literature exists discussing potential clinical trial endpoints (Schmetterer et al., 2023), there is a paucity of data regarding the ocular surface. A variety of disease biomarkers such as ocular surface staining, tear film osmolarity, noninvasive tear break up time (NIBUT) and others have been proposed in the past as suitable outcome parameters to assess therapeutic success (Wolffsohn et al., 2017). These biomarkers are used from early clinical trials to provide proof of concept (clinical phase II) to pivotal and post‐marketing trials to assess the clinical benefit of the therapeutic intervention (clinical phases III and IV) (Novack et al., 2017). In addition, for regulatory approval, a clinically meaningful benefit is required in terms of both clinical signs and patient‐reported outcomes (symptoms) (Novack et al., 2017; US Food & Drug Administration, 2020).

This systematic review analyzes the endpoints used in controlled clinical trials. For this purpose, published clinical trials in the field of DED were sampled from 2000 to 2023 and the used clinical endpoints were assessed. Based on these data, the different outcome parameters of clinical trials and their frequency of use are discussed in the context of clinical trials and regulatory approval.

## 2 METHODS

### 2.1 Study registration

The present systematic review was registered with the International Prospective Register of Systematic Reviews (PROSPERO; registration number: CRD42022350817) and adhered to the Preferred Reporting Items for Systematic Reviews and Meta‐analyses (Page et al., 2021).

### 2.2 Search strategy

The following databases were searched for studies published between 1 January 2000 and 31 December 2023: PubMed, Embase, The Cochrane Library, Web of Science and Medline. Combinations of the following terms were used for the searches: (‘Dry Eye’ OR ‘DED’ OR ‘Ocular Surface Disease’ OR ‘OSD’ OR ‘Keratoconjunctivitis sicca’) AND (‘Therapy’ OR ‘Treatment’ OR ‘Eye Drops’ OR ‘Efficacy’). We decided to include studies from the last 20 years as this would provide an objective overview of commonly used endpoints in DED studies and also include newer methods such as NIBUT.

### 2.3 Eligibility criteria

In Table 1, inclusion and exclusion criteria for studies are presented. In addition, only studies in the English language were selected.

**TABLE 1:** Inclusion and exclusion criteria for selection of studies to be included in the present systematic review.

Inclusion criteria	Exclusion criteria
Longitudinal studies	Non‐human studies
Controlled studies	Studies in healthy subjects
Included patients with dry eye or keratoconjunctivitis sicca or ocular surface disease	Case reports or case series
At least 2 time points for assessment of the primary endpoint	Reviews
At least 20 participants per group	Meta‐analyses
Participants had to be at least 20 years old	Pooled analyses
	Cross‐sectional studies
	Meeting abstracts
	Retrospective studies
	Studies in contact lens wearers
	Studies in patients with secondary dry eye (i.e. after surgery)
	Studies focusing explicitly on MGD, Sjögren or GVHD

Abbreviations: GVHD, graft versus host disease; MGD, meibomian gland disease.

### 2.4 Study selection

As first step, database search for the keywords listed above was performed. All search results were then downloaded and imported into the online application Rayyan (https://www.rayyan.ai/), a software that is specifically designed for the conduction of systematic reviews, and duplicates were removed (Ouzzani et al., 2016). For identical references, this was done automatically by Rayyan, for possible duplicates, this was done by the two reviewers (LP, VP) manually by screening through the suggestions offered by the software. All remaining abstracts were then independently screened for eligibility by the two reviewers via Rayyan. If discrepancies between reviewer selections occurred, these were resolved by the decision of a third independent reviewer (DS). The full‐texts of the studies selected by Title/Abstract were imported into the software and then underwent a full‐text review by the two independent reviewers. Again, discrepancies between reviewers' selections were resolved by the decision of the third independent reviewer. Table 2 lists the data that were extracted from each full‐text article for the present review.

**TABLE 2:** Listing of variables that were extracted from included full‐text articles and description of labels for variables.

Variable	Type of measurement	Value labels
Number of patients	Metric	N/A
Number of study groups	Metric	N/A
Study phase (most accurate)	Ordinal	2, 3, 4
Experimental arm	Nominal	N/A
Type of experimental arm	Nominal	Topical medication,^a^ topical lubricant,^a^ systemic medication, nutritional supplement, medical device therapy, surgical intervention
Control arm 1	Nominal	N/A
Type of control 1	Nominal	Placebo, active, no intervention
Control arm 2	Nominal	N/A
Type of control 2	Nominal	Placebo, active, no intervention
Primary objective outcome parameter	Nominal	TBUT, NIBUT, corneal fluorescein staining, ocular surface staining (other than fluorescein),^b^ Schirmer I, Schirmer II, tear film osmolarity, other,^c^ none
Primary subjective outcome parameter	Nominal	OSDI, IDEEL, SPEED, SANDE, VAS, EDS, other,^c^ none
Time to assessment of outcome in weeks	Metric	N/A
Objective outcome significant vs. control	Dichotomous	Yes, no
Objective outcome significant vs. baseline	Dichotomous	Yes, no
Subjective outcome significant vs. control	Dichotomous	Yes, no
Subjective outcome significant vs. baseline	Dichotomous	Yes, no

Abbreviations: EDS, Eye Dryness Score; IDEEL, Impact of Dry Eye on Everyday Life; NIBUT, non‐invasive tear film break up time; OSDI, Ocular Surface Disease Index; SANDE, Symptom Assessment Questionnaire in Dry Eye; SPEED, Standard Patient Evaluation of Eye Dryness Questionnaire; TBUT, tear film break up time; VAS, Visual Analogue Scale.

^a^ Topical medication includes all eye drops containing a medicinal product (i.e. cyclosporine, corticosteroids), while topical lubricants refer to all eye drops that are not considered a pharmacotherapy in its classical sense (i.e. artificial tears containing povidone, hyaluronic acid).

^b^ Refers to conjunctival staining, lissamine green or rose Bengal staining.

^c^ Refers to all outcomes that did not fall within the predefined variables.

### 2.5 Quality assurance

Study quality was assessed using the Delphi list (Verhagen et al., 1998). Only studies reaching a score >5 were included in the final analysis.

### 2.6 Data analysis

All data extracted from the articles according to Table 2 were entered into an SPSS file (IBM SPSS Statistics Version 28.0.1.0, Armonk, USA). Only descriptive statistics were used to report outcomes and no inferential statistics were performed. In particular, crosstabs were created to report frequencies and percentages for objective and subjective outcome parameters and to identify the number of trials that reported significant changes versus baseline or comparator or whether the noninferiority margin was reached.

## 3 RESULTS

### 3.1 Literature search

**FIGURE 1:**
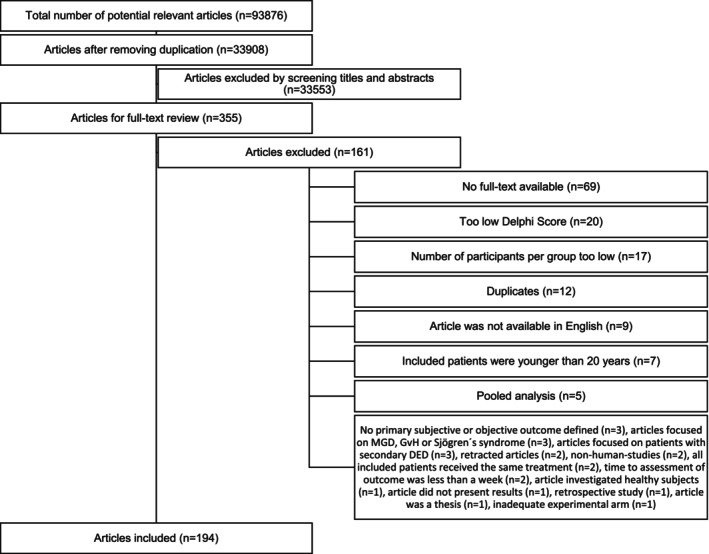
Study flow diagram.

A study flow diagram is presented in Figure 1. At the initial search, a total of 93 876 articles was found, of which 33 908 remained after duplicates were removed. After screening through the abstracts, 355 articles remained for full‐text review, of which 194 were included in the present systematic review. 161 articles were removed after full‐text analysis because of the following reasons: no full‐text available (*n* = 69), Delphi‐Score ≤5 (*n* = 20), number of participants per group did not meet the inclusion criteria (*n* = 17), articles were duplicates (*n* = 12), article was not available in English (*n* = 9), included patients were younger than 20 years (*n* = 7), pooled‐analysis (*n* = 5), articles had no primary subjective or objective outcome (*n* = 3), articles focused either on MGD, GvH or Sjögren (*n* = 3), articles focused on patients with secondary DED (*n* = 3), article was retracted (*n* = 2), non‐human studies (*n* = 2), all included patients received the same treatment (*n* = 2), time to assessment of outcome was less than a week (*n* = 2), article investigated healthy subjects (*n* = 1), article did not present results (*n* = 1), article was a retrospective study (*n* = 1), article was a thesis (*n* = 1), article had an inadequate experimental arm (*n* = 1). Details on the included studies are provided in Table S1.

### 3.2 General description of the included studies

**FIGURE 2:**
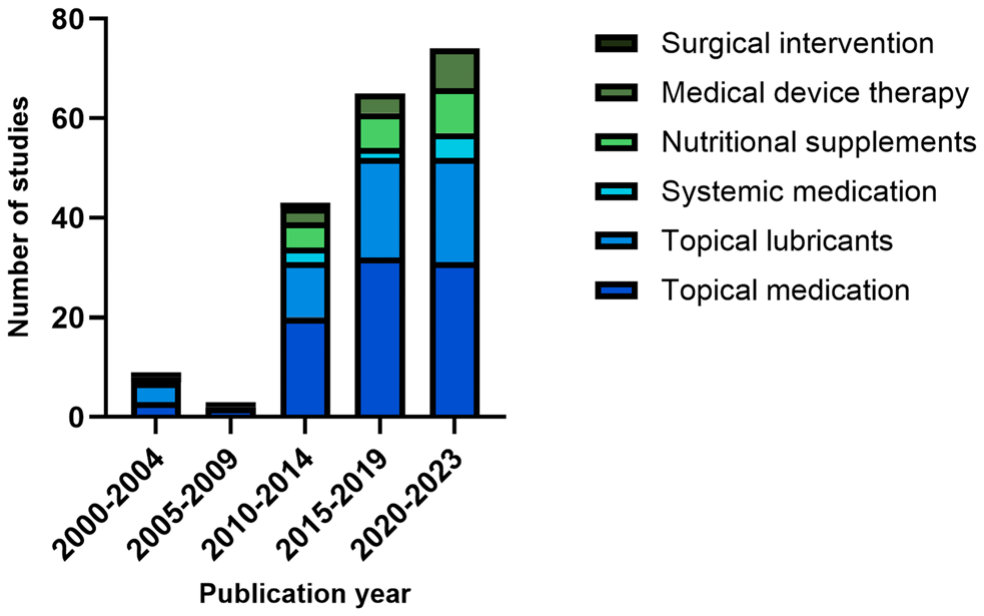
Number of articles published per 5‐year interval broken down by type of intervention.

All studies included in the present review were controlled (Placebo: 59 studies, active control: 135 studies). The most frequently investigated product was topical medication defined as a medicinal product with a pharmacological active ingredient (88 studies), followed by topical lubricants (57 studies), nutritional supplements (22 studies), medical device therapy (16 studies), systemic medication (10 studies) and surgical intervention (1 study). As shown in Figure 2, the number of articles published constantly increased over the years.

The majority of studies were phase 2 (*n* = 37) and phase 3 (*n* = 35), while only 13 studies were phase 4. For 109 studies, no study phase was reported. The median number of study participants was 100 patients and the median study duration until outcome assessment was 12 weeks. The median Delphi score was 7 points. 17.6% of the studies received 6 points, 36.3% received 7 points, 34.2% received 8 points and 11.9% received 9 points on the Delphi list by the graders. Studies with a Delphi score ≤5 were not included.

### 3.3 Objective outcome parameters

The most frequently used primary objective outcome parameter was corneal fluorescein staining (45 studies) followed by TBUT (41 studies) and ocular surface staining (other than fluorescein; 26 studies). An overview is provided in Table 3. The type of scoring system used to report corneal fluorescein staining was heterogeneous, with the National Eye Institute scale being the most commonly used (*n* = 15), followed by the Oxford or modified Oxford scale (*n* = 12), inferior corneal staining (*n* = 7) and the Schimmura method (*n* = 5). Other scoring systems such as the van Bijsterveld score or study‐specific scores were also used in single studies.

**TABLE 3:** Objective and subjective primary outcome parameters in clinical studies (*n* = 194).

Outcome parameter	Frequency	Percent
Objective outcome parameters		
Corneal fluorescein staining	45	23.2
TBUT	41	21.1
Ocular surface staining (other than fluorescein)	26	13.4
Schirmer I	14	7.2
Schirmer II	9	4.6
NIBUT	8	4.1
Tear film osmolarity	5	2.6
Other	24	12.4
No objective outcome defined	22	11.3
Subjective outcome parameters		
OSDI	96	49.5
VAS	14	7.2
EDS	5	2.6
SANDE	5	2.6
SPEED	5	2.6
IDEEL	3	1.5
Other	60	30.9
No subjective outcome defined	6	3.1

Abbreviations: EDS, Eye Dryness Score; IDEEL, Impact of Dry Eye on Everyday Life; NIBUT, non‐invasive tear film break‐up time; OSDI, Ocular Surface Disease Index; SANDE, Symptom Assessment Questionnaire in Dry Eye; SPEED, Standard Patient Evaluation of Eye Dryness Questionnaire; TBUT, tear film break‐up time; VAS, Visual Analog Scale.

**FIGURE 3:**
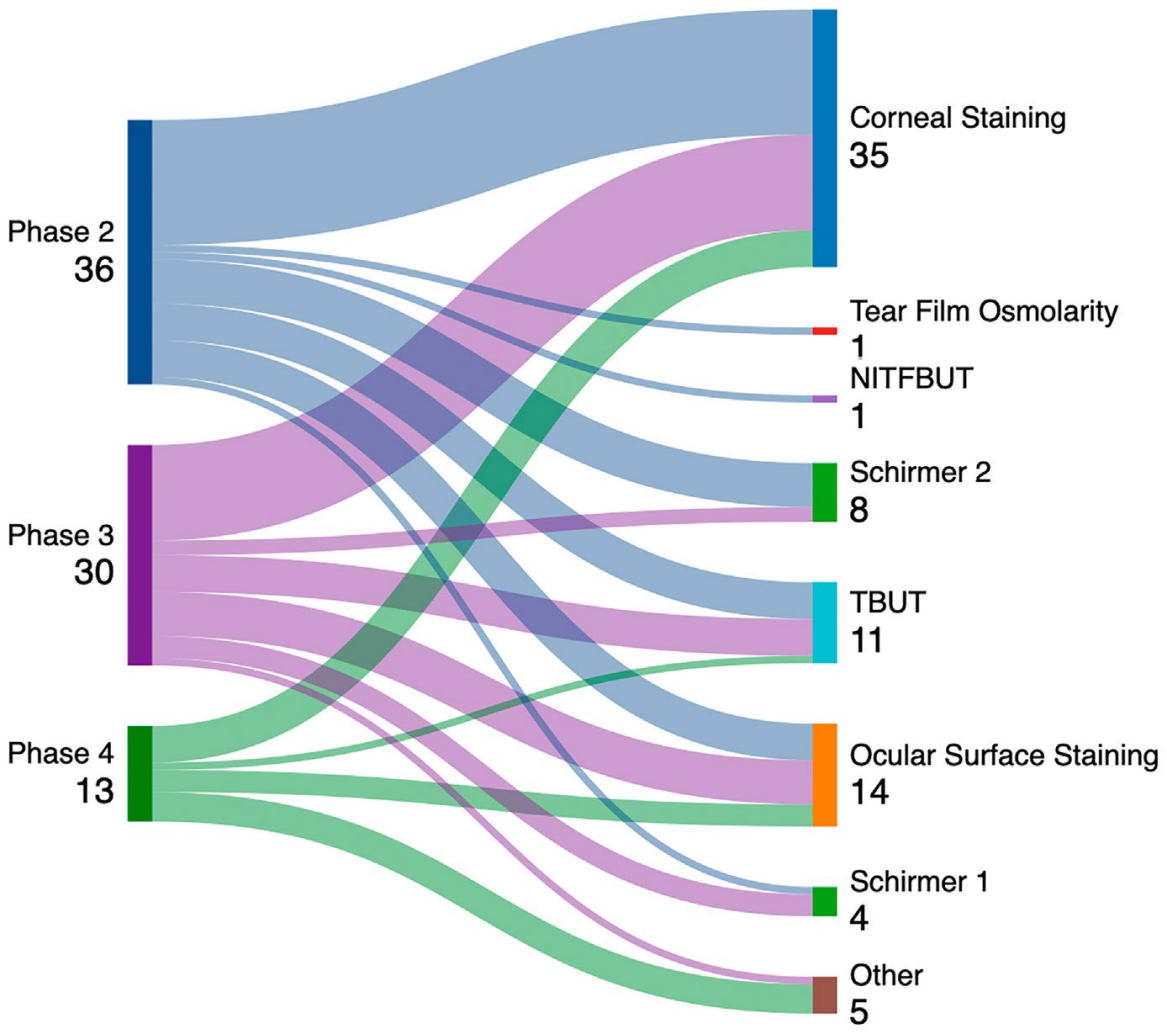
Objective primary outcomes for different clinical phases (2–4). The numbers on the left side refer to the number of studies included in the analysis per phase. The numbers on the right side refer to the number of studies that investigated the respective endpoint. NIBUT, non‐invasive tear film break up time; TBUT, tear film break up time.

Figure 3 shows objective primary outcome parameters dependent on the clinical phase of the study.

A significant improvement in the primary objective outcome parameter versus baseline value was found in 103 studies (53.1%), while no significant improvement was reported in 11 studies (5.7%). For 80 studies (41.2%), no statistics versus baseline are reported.

A significant improvement in the primary objective outcome parameter versus control was reported in 91 studies (46.9%), while no significant difference was reported in 58 studies (29.9%). In 15 studies (7.7%), the noninferiority margin was reached, while no outcome was reported in 30 studies (15.5%). Table 4 shows which objective outcome parameters were statistically significant versus baseline and which were statistically significant versus control.

**TABLE 4:** Objective primary outcome parameters that improved significantly versus control and significantly different versus baseline.

Outcome parameter	Significant vs. baseline	No change vs. baseline	Significant vs. control	No difference vs. control	Noninferiority margin reached
Corneal fluorescein staining	21	1	23	19	3
TBUT	31	4	22	17	2
Ocular surface staining (other than fluorescein)	12	0	11	6	9
Schirmer I	10	2	7	5	1
Schirmer II	5	0	5	3	0
Tear film osmolarity	1	1	5	0	0
NIBUT	4	1	4	3	0
Other	19	2	14	5	0

Abbreviations: NIBUT, non‐invasive tear film break‐up time; TBUT, tear film break‐up time.

### 3.4 Subjective outcome parameters

The most frequently used primary subjective outcome parameter was the Ocular Surface Disease Index (OSDI, 96 studies) followed by the use of Visual Analogue Scales (VAS, 14 studies). An overview is provided in Table 3.

**FIGURE 4:**
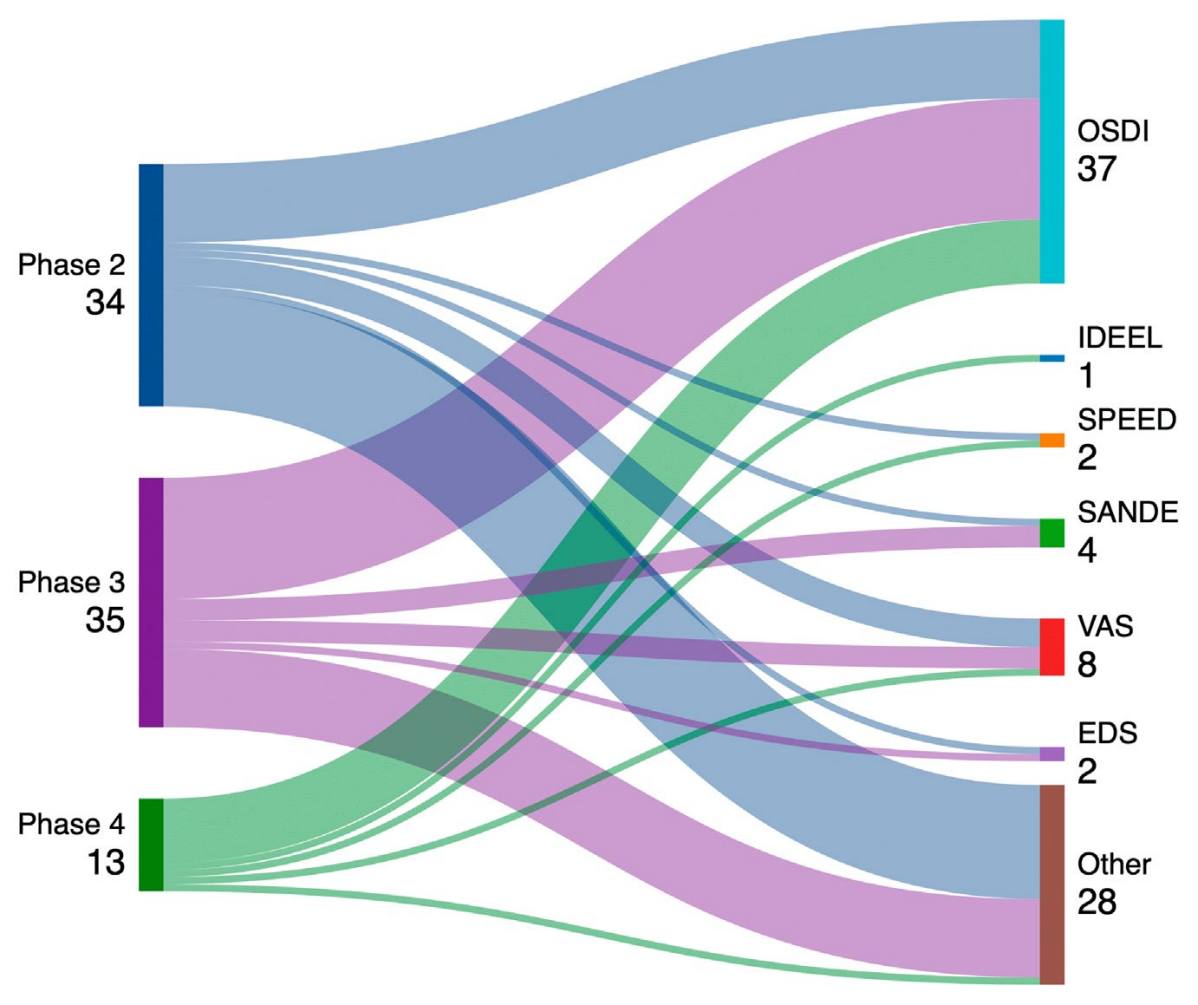
Subjective primary outcomes for different clinical phases (2–4). The numbers on the left side refer to the number of studies included in the analysis per phase. The numbers on the right side refer to the number of studies that investigated the respective endpoint. EDS, Eye Dryness Score; IDEEL, Impact of Dry Eye on Everyday Life; OSDI, Ocular Surface Disease Index; SANDE, Symptom Assessment Questionnaire in Dry Eye; SPEED, Standard Patient Evaluation of Eye Dryness Questionnaire; VAS, Visual Analogue Scale.

Figure 4 represents a breakdown analysis of the different primary subjective outcome parameters based on the clinical phase of the study.

A significant improvement in the primary subjective outcome parameter versus baseline value was found in 108 studies (55.7%), while no significant improvement was reported in 10 studies (5.2%). For 76 studies (39.2%), no statistics versus baseline are reported.

A significant improvement in the primary subjective outcome parameter versus control was reported in 87 studies (44.8%), while no significant difference was reported in 83 studies (42.8%). In 5 studies (2.6%), the noninferiority margin was reached and in 1 study it was not reached (0.5%), while no outcome was reported for 18 studies (9.3%). Table 5 shows which subjective outcome parameters were statistically significant versus baseline and which were statistically significant versus control.

**TABLE 5:** Subjective primary outcome parameters that turned out significantly different versus baseline and that improved significantly versus control.

Outcome parameter	Significant vs. baseline	No change vs. baseline	Significant vs. control	No difference vs. control	Noninferiority margin reached	Noninferiority margin not reached
OSDI	68	4	46	40	5	0
VAS	3	1	3	10	0	1
EDS	2	1	2	2	0	0
SANDE	4	0	1	3	0	0
SPEED	4	0	1	3	0	0
IDEEL	0	0	1	2	0	0
Other	27	4	33	23	0	0

Abbreviations: EDS, Eye Dryness Score; IDEEL, Impact of Dry Eye on Everyday Life; OSDI, Ocular Surface Disease Index; SANDE, Symptom Assessment Questionnaire in Dry Eye; SPEED, Standard Patient Evaluation of Eye Dryness Questionnaire; VAS, Visual Analogue Scale.

### 3.5 Outcome parameters regarding type of intervention

Table 6 shows how many findings turned out to be statistically significant regarding the type of intervention used.

**TABLE 6:** Statistically significant findings regarding the used intervention.

Type of intervention	Objective outcome parameter		Subjective outcome parameter	
	Significant vs. baseline (year/*n*)	Significant vs. control (year/*n*)	Significant vs. baseline (year/*n*)	Significant vs. control (year/*n*)
Topical medication	48/2	50/24	49/3	45/37
Topical lubricants	30/6	18/23	35/2	19/27
Nutritional supplements	13/1	9/5	11/2	11/6
Medical device therapy	9/0	10/3	9/0	10/6
Systemic medication	3/2	4/3	4/3	2/6

## 4 DISCUSSION

The number of articles published in the field of DED research has increased steadily over the past decades. In the period from 1 January 2000 to 31 December 2023, we identified a total number of 33 908 articles matching the primary search criteria for DED. As DED is an umbrella term representing a spectrum of diseases and therapy varies widely depending on pathogenesis and severity, it is not surprising that clinical trials include a variety of different approaches. According to the numbers, among the analysed studies that met the inclusion/exclusion criteria, the most common were drug studies (88 studies), followed by medical device studies for topical lubricants (57 studies), dietary supplements (22 studies), other non‐topical medical device studies (16 studies), systemically applied drugs (10 studies) and surgical interventions (1 study). The number of trials matching our inclusion criteria has also significantly increased over the years. While we identified 9 articles for the period from 2000 to 2004, there were 74 articles that could be included for the period from 2020 to 2023.

These data highlight a common problem in the treatment of DED: Over‐the‐counter (OTC) medical devices are widely used to treat early to moderate DED, significantly surpassing the use of prescription medications for severe forms of the disease. However, there is a notable disparity between the extensive data available for pharmaceuticals and the limited evidence for OTC topical medical devices. Approved pharmacologic treatments benefit from robust data from large, randomized controlled trials that provide high‐quality evidence of efficacy, safety and comparative effectiveness. In contrast, OTC products often lack such rigorous data, resulting in a significant evidence gap. This lack of comprehensive efficacy and comparative studies complicates informed decision‐making for the use of medical devices in DED and hinders the development of standardized treatment protocols. As an exception, medical devices, herein referred to as ‘topical lubricants’ such as hyaluronic acid‐based artificial tears are usually registered as drugs in some countries and therefore undergo more extensive research before marketing approval.

Although there are differences between regulatory agencies regarding the requirements for approval of pharmacological treatments for DED, it is generally imperative to demonstrate efficacy by demonstrating both objective signs and subjective symptoms (US Food & Drug Administration, 2020). Objective signs include measurable clinical parameters such as tear break‐up time (TBUT), corneal and conjunctival staining scores and Schirmer test results, which indicate functional and structural integrity of the ocular surface and tear production (Wolffsohn et al., 2017). Our data show that the most commonly used primary objective outcome parameter was corneal fluorescein staining which was used together with other ocular surface staining methods in approximately one third of all studies, followed by TBUT and Schirmer's test (Table 3). Although ocular surface staining, decreased TBUT and Schirmer's test are all accepted as primary endpoints, our data indicate that corneal fluorescein staining is one of the most stable endpoints and is therefore often selected as an outcome, especially for phase III clinical trials (Figure 2). TBUT and Schirmer's tests do not have generally accepted cut‐off values or differences that are considered clinically significant, which limits their general applicability. For example, TBUT is considered pathological at 10 s or less in the US and EU, while Japanese authorities require a more stringent cut‐off of 5 s or less. As for the Schirmer test, the Food and Drug Administration (FDA) usually considers a 10‐mm wetting or more in the Schirmer test score as sufficient (US Food & Drug Administration, 2020), while EU regulators have expressed doubt that a 10 mm difference is clinically relevant (EMA CHMP, 2018). As there are currently no official guidelines for the development of DED drugs in the EU, the acceptability of primary and key secondary endpoints needs to be clarified through scientific advice procedures or comparable advisory procedures before the study.

Of note, our study shows that for the most frequently measured parameter (corneal fluorescein staining), a large number of studies failed to reach statistical significance versus control (Table 4). This is a well‐known challenge in DED research and may be at least partially related to the fact that the control substance itself, even if just the vehicle for an active treatment is used, induces beneficial effects on the ocular surface and therefore partially masks the treatment effect (Novack et al., 2017). This creates the awkward situation that the better the ocular surface tolerability of the vehicle, the more difficult it is to demonstrate superiority of the active treatment. In principle, an active control as standard of care could be an option to avoid this situation; however, the FDA guidance document clearly states that equivalence or non‐inferiority trials are not recommended in the field of DED (US Food & Drug Administration, 2020). This is also reflected in the results of the present study, where non‐inferiority studies are only a small minority. The Tear Film and Ocular Surface Society International Dry Eye Workshop (DEWS) recommends a study design in which treatment initiation is masked to both the investigator and the subject, or alternatively, a withdrawal design in which all subjects receive an initial active treatment followed by final randomization to vehicle or drug treatment (Novack et al., 2017). However, the latter approach usually needs higher sample sizes and also has the risk of carry‐over effects from the initial treatment phase, which considerably complicates the trial design and increases the costs.

In addition to clinical signs, regulatory agencies require that clinical trials demonstrate meaningful improvements in patients' symptoms to ensure that the therapeutic intervention not only addresses the physiological aspects of the disease but also improves patients' overall quality of life. In this regard, our data show that the OSDI is the most widely used symptom assessment test, with nearly half of the trials using the OSDI as the primary outcome measure (Table 3). The OSDI is a 12‐item questionnaire that provides an estimate of both DED‐related symptoms and vision‐related functioning. The OSDI has undergone extensive validity and reliability testing and has demonstrated excellent test–retest reliability and good validity (Grubbs et al., 2014; Guillemin et al., 2012; Schiffman et al., 2000). Importantly, a minimal clinically important difference has been proposed and generally accepted for the OSDI for both mild to moderate disease and severe disease (Grubbs et al., 2014). More recently, visual analogue scales have gained interest in the assessment of DED‐related symptoms. This method has been used to assess symptoms in several diseases (Miller & Ferris, 1993) and involves a simple 100 mm long line on which patients mark their perceived severity of symptoms such as dryness, discomfort, visual disturbance and others (McCormack et al., 1988; McMonnies, 2016). The VAS is particularly valuable in the management of DED due to its sensitivity to small changes in symptom severity and has been used in recent Phase III regulatory trials (McMonnies, 2016; Peng et al., 2024). The ‘Symptom Assessment in Dry Eye’ (SANDE) is based on a visual analogue scale and consists of two questions assessing the severity and frequency of dry eye syndrome (Schaumberg et al. 2007), SANDE, Impact of Dry Eye on Everyday Life (IDEEL), Standard Patient Evaluation of Eye Dryness Questionnaire (SPEED) and Eye Dryness Score (EDS), are, however, less frequently used (Table 3). For symptoms, our analysis shows that many of the trials failed to reach a statistically significant difference compared to control (Table 5). Possible reasons for this failure have been discussed earlier with respect to clinical signs and may also apply to the assessment of clinical symptoms. However, most studies report a significant difference from baseline, indicating a treatment effect of the tested agents (Table 5).

Our analysis reveals persistent limitations in the current landscape of clinical trial design in DED—mainly, the lack of consensus on the most reliable and clinically meaningful outcome parameters. Despite the frequent use of corneal fluorescein staining and the OSDI as primary endpoints, the high number of negative trials raises questions regarding the sensitivity of these endpoints to detect therapeutic benefit. Invasive techniques such as fluorescein staining can also interfere with the ocular surface, potentially altering subsequent test outcomes and introducing variability. Thus, concerns have been raised in respect to their use and current recommendations from expert bodies suggest the use of non‐invasive, more reproducible approaches such as NIBUT (Wolffsohn et al., 2017). It may be speculated that the high frequency of use of the above‐mentioned invasive tests in clinical trials may stem from efforts to maintain comparability with earlier studies and reflect regulatory precedent, rather than evidence of their clinical superiority compared to newer, non‐invasive methods. A better understanding of disease pathophysiology and more data on new non‐invasive testing methods may, in future, facilitate the development of validated biomarkers that are more sensitive to therapeutic effects (Roy et al., 2017).

Despite the comprehensive nature of this review, several limitations must be acknowledged to provide a balanced perspective on the findings. A well‐known limitation of synthesizing the existing literature is the potential for publication bias (Easterbrook et al., 1991). This bias can occur because studies with positive or significant results are published more often or earlier than those with negative or null results. As a result, positive outcomes may be disproportionately represented in the literature, biasing the overall impression toward positive outcomes (Easterbrook et al., 1991). Another potential limitation is the incomplete reporting found in some publications. Some studies do not provide complete information, such as the clinical phase of the study or the detailed methodology. To overcome this problem, we tried to contact the corresponding authors. If this approach was unsuccessful, the parameter was classified as ‘not available’.

## 5 CONCLUSION

Our analysis revealed corneal fluorescein staining as the most frequent primary endpoint for assessing clinical signs of DED, while the OSDI and visual analogue scale were the predominant choices for symptom evaluation. Notably, a substantial proportion of studies failed to demonstrate statistically significant differences between treatment and control groups, despite improvements from baseline. This reflects the challenges in endpoint selection due to the multifactorial nature of DED and the variability in patient symptoms and response to treatment. To enhance the efficiency and reliability of DED clinical trials, a consensus on optimal outcome measures is crucial, and the exploration of novel endpoints should be prioritized.

## AUTHOR CONTRIBUTIONS

VP: Data curation, investigation, writing—original draft, visualization; DS: Conceptualization, methodology, writing—original draft, supervision; LP: Data curation, investigation; PJ: Data curation, investigation; TL: Writing—review and editing; UG: Data curation, investigation; JC: Writing—review and editing; LS: Writing—review and editing; GG: Conceptualization, methodology, writing—original draft, supervision.

## Supporting information


Data S1.
