# Supplementary material for: Outcome parameters in studies investigating dry eye disease: A systematic literature review
Source: Acta Ophthalmol. 2025 Nov 20;104(4):e363–72. doi: 10.1111/aos.70031 (PMC13166401; doi:10.1111/aos.70031)
Supplement: Supplementary file 1 — Data S1. [file AOS-104-e363-s001.docx]

| **Author** | **Number of patients** | **Number of groups** | **Experimental arm** | **Control arm 1** | **Control arm 2** | **Control arm 3** | **Primary objective outcome** | **Primary subjective outcome** |
| --- | --- | --- | --- | --- | --- | --- | --- | --- |
| Aguilar et al. 2014 | 51 | 2 | Topical lubricant | Placebo | N/A | N/A | NITFBUT | Not defined |
| Akpek et al. 2023 | 834 | 2 | Topical medication | Placebo | N/A | N/A | Corneal fluorescein staining | VAS |
| Aragona et al. 2020 | 454 | 2 | Topical lubricant | Active | N/A | N/A | TFBUT | OSDI |
| Aragona et al. 2002 | 86 | 2 | Topical lubricant | Placebo | N/A | N/A | Other | VAS |
| Asbell et al. 2018 | 101 | 2 | Topical lubricant | Active | N/A | N/A | Ocular surface staining | IDEEL |
| Asbell et al. 2018 | 535 | 2 | Nutritional supplements | Placebo | N/A | N/A | Not defined | OSDI |
| Avni et al. 2010 | 80 | 2 | Systemic medication | Placebo | N/A | N/A | TFBUT | Other |
| Ayres et al. 2023 | 345 | 2 | Medical device therapy | Active | N/A | N/A | TFBUT | OSDI |
| Baiza-Durán et al. 2010 | 183 | 3 | Topical medication | Active | Placebo | N/A | Not defined | Other |
| Baiza-Durán et al. 2023 | 101 | 2 | Topical lubricant | Active | N/A | N/A | TFBUT | OSDI |
| Barabino et al. 2014 | 48 | 2 | Topical lubricant | Active | N/A | N/A | TFBUT | OSDI |
| Baudouin et al. 2012 | 82 | 2 | Topical lubricant | Active | N/A | N/A | Ocular surface staining | OSDI |
| Baudouin et al. 2017 | 177 | 2 | Topical medication | Placebo | N/A | N/A | Corneal fluorescein staining | OSDI |
| Baudouin et al. 2017 | 495 | 2 | Topical medication | Placebo | N/A | N/A | Corneal fluorescein staining | VAS |
| Belalcázar-Rey et al. 2021 | 326 | 3 | Topical lubricant | Active | Active | N/A | Other | OSDI |
| Benelli et al. 2010 | 60 | 3 | Topical lubricant | Active | Active | N/A | Tear film osmolarity | Not defined |
| Bhargava et al. 2015 | 456 | 2 | Nutritional supplements | Placebo | N/A | N/A | Not defined | Other |
| Bhargava et al. 2023 | 950 | 2 | Nutritional supplements | Placebo | N/A | N/A | Other | Other |
| Bing Zhang et al. 2019 | 60 | 2 | Topical medication | Active | N/A | N/A | Other | Other |
| Brar et al. 2021 | 60 | 2 | Topical lubricant | Active | N/A | N/A | Other | SPEED |
| Brignole-Baudouin et al. 2011 | 138 | 2 | Nutritional supplements | Placebo | N/A | N/A | Other | Other |
| Brignole et al. 2001 | 169 | 3 | Topical medication | Active | Placebo | N/A | Other | Not defined |
| Brzheskiy et al. 2015 | 240 | 2 | Topical medication | Placebo | N/A | N/A | Schirmer I | Other |
| Byun et al. 2012 | 44 | 2 | Topical medication | Active | N/A | N/A | Not defined | Other |
| Cai and Zhang 2020 | 52 | 2 | Medical device therapy | Active | N/A | N/A | TFBUT | OSDI |
| Calonge et al. 2023 | 81 | 2 | Topical lubricants | Active | N/A | N/A | Ocular surface staining | Other |
| Carreira et al. 2023 | 39 | 2 | Topical lubricants | Active | N/A | N/A | TFBUT | OSDI |
| Chen et al. 2019 | 240 | 2 | Topical medication | Placebo | N/A | N/A | Other | Other |
| Chen et al. 2014 | 115 | 2 | Topical medication | Active | N/A | N/A | Not defined | Other |
| Chen et al. 2023 | 66 | 3 | Topical medication | Active | Placebo | N/A | NITFBUT | OSDI |
| Chen et al. 2010 | 233 | 2 | Topical medication | Placebo | N/A | N/A | Other | Other |
| Chiambaretta et al. 2017 | 105 | 2 | Topical lubricant | Active | N/A | N/A | Ocular surface staining | OSDI |
| Cho et al. 2013 | 85 | 4 | Topical medication | Active | Active | Active | Not defined | OSDI |
| Choi et al. 2015 | 50 | 2 | Medical device therapy | Placebo | N/A | N/A | TFBUT | OSDI |
| Christensen et al. 2004 | 87 | 2 | Topical lubricant | Active | N/A | N/A | Ocular surface staining | VAS |
| Chung et al. 2016 | 412 | 2 | Topical lubricant | Active | N/A | N/A | Not defined | Other |
| Clark et al. 2021 | 300 | 3 | Topical medication | Active | Placebo | N/A | Ocular surface staining | Other |
| Cohen et al. 2014 | 147 | 2 | Topical lubricant | Active | N/A | N/A | Corneal fluorescein staining | Other |
| Cohn et al. 2019 | 61 | 2 | Medical device therapy | Placebo | N/A | N/A | Schirmer II | OSDI |
| Craig et al. 2021 | 99 | 2 | Topical lubricant | Active | N/A | N/A | NITFBUT | OSDI |
| Davitt et al. 2010 | 105 | 2 | Topical lubricant | Active | N/A | N/A | Ocular surface staining | Other |
| Deinema et al. 2017 | 60 | 3 | Nutritional supplements | Active | Placebo | N/A | Tear film osmolarity | OSDI |
| Dong et al. 2022 | 100 | 2 | Topical medication | Placebo | N/A | N/A | Corneal fluorescein staining | Other |
| Downie et al. 2020 | 242 | 2 | Topical lubricant | Active | N/A | N/A | TFBUT | OSDI |
| Eom et al. 2023 | 222 | 3 | Topical medication | Active | Placebo | N/A | TFBUT | OSDI |
| Eom and Kim 2021 | 450 | 3 | Topical medication | Active | Active | N/A | TFBUT | OSDI |
| Eom et al. 2023 | 178 | 2 | Topical medication | Active | N/A | N/A | Schirmer I | OSDI |
| Epitropoulos et al. 2016 | 105 | 2 | Nutritional supplements | Placebo | N/A | N/A | Tear film osmolarity | OSDI |
| Essa et al. 2018 | 50 | 4 | Topical lubricant | Active | Active | Active | NITFBUT | OSDI |
| Evans et al. 2023 | 160 | 2 | Topical medication | Placebo | N/A | N/A | Corneal fluorescein staining | Other |
| Fan et al. 2023 | 108 | 2 | Nutritional supplements | Placebo | N/A | N/A | Schirmer I | OSDI |
| Fondi et al. 2018 | 40 | 2 | Topical lubricant | Active | N/A | N/A | TFBUT | OSDI |
| García-Conca et al. 2019 | 83 | 2 | Topical medication | Active | N/A | N/A | Not defined | OSDI |
| Goldberg et al. 2019 | 744 | 2 | Topical medication | Placebo | N/A | N/A | Schirmer I | Other |
| Goldstein et al. 2017 | 74 | 3 | Topical medication | Active | Placebo | N/A | Corneal fluorescein staining | OSDI |
| Gong et al. 2015 | 497 | 2 | Topical medication | Active | N/A | N/A | Ocular surface staining | Other |
| Groß et al. 2017 | 70 | 2 | Topical lubricant | Active | N/A | N/A | Ocular surface staining | Other |
| Groß et al. 2018 | 80 | 2 | Topical lubricant | Active | N/A | N/A | Ocular surface staining | Other |
| Grosskreutz et al. 2015 | 72 | 3 | Systemic medication | Active | Placebo | N/A | Corneal fluorescein staining | OSDI |
| Haji-Ali-Nili et al. 2019 | 60 | 2 | Topical lubricant | Placebo | N/A | N/A | NITFBUT | OSDI |
| Hassan et al. 2022 | 60 | 2 | Topical medication | Active | N/A | N/A | Corneal fluorescein staining | OSDI |
| Holland et al. 2017 | 711 | 2 | Topical medication | Placebo | N/A | N/A | Not defined | EDS |
| Hori et al. 2022 | 337 | 2 | Topical medication | Placebo | N/A | N/A | Corneal fluorescein staining | Other |
| Hovanesian et al. 2022 | 50 | 2 | Topical medication | Active | N/A | N/A | Corneal fluorescein staining | SPEED |
| Hu et al. 2021 | 59 | 2 | Medical device therapy | Active+Placebo | N/A | N/A | TFBUT | OSDI |
| Huang et al. 2016 | 66 | 2 | Nutritional supplements | Placebo | N/A | N/A | TFBUT | Other |
| Hussain et al. 2020 | 43 | 2 | Nutritional supplements | Placebo | N/A | N/A | Ocular surface staining | OSDI |
| Hwang et al. 2014 | 150 | 3 | Topical medication | Active | Active | N/A | TFBUT | OSDI |
| Iester et al. 2000 | 135 | 2 | Topical lubricant | Active | N/A | N/A | Schirmer I | Other |
| Jackson et al. 2011 | 43 | 2 | Nutritional supplements | Active | N/A | N/A | TFBUT | Other |
| Järvinen et al. 2011 | 100 | 2 | Nutritional supplements | Placebo | N/A | N/A | Other | Other |
| Jee et al. 2014 | 100 | 2 | Topical medication | Active | N/A | N/A | Not defined | OSDI |
| Jerkins et al. 2020 | 231 | 2 | Topical lubricant | Active | N/A | N/A | TFBUT | VAS |
| Jiang et al. 2017 | 60 | 2 | Topical lubricant | Placebo | N/A | N/A | Other | OSDI |
| Jing Yu 2016 | 60 | 2 | Topical medication | Active | N/A | N/A | Other | Other |
| Jun et al. 2022 | 82 | 3 | Topical lubricant | Active | Active | N/A | Corneal fluorescein staining | OSDI |
| Jung et al. 2023 | 80 | 3 | Topical medication | Active | Active | N/A | Corneal fluorescein staining | SANDE |
| Kaercher et al. 2022 | 80 | 2 | Topical lubricant | Active | N/A | N/A | TFBUT | OSDI |
| Kaido et al. 2012 | 43 | 2 | Surgical intervention | Active | N/A | N/A | Not defined | Other |
| Kallab et al. 2020 | 60 | 2 | Topical medication | Active | N/A | N/A | Other | OSDI |
| Kan et al. 2020 | 360 | 4 | Nutritional supplements | Active | Active | Placebo | Other | Other |
| Kangari et al. 2013 | 64 | 2 | Nutritional supplements | Placebo | N/A | N/A | TFBUT | OSDI |
| Kawakita et al. 2016 | 65 | 2 | Topical medication | Placebo | N/A | N/A | Corneal fluorescein staining | Other |
| Kim et al. 2009 | 150 | 3 | Topical medication | Active | Active | N/A | Not defined | OSDI |
| Kim et al. 2017 | 101 | 2 | Topical medication | Active | N/A | N/A | Corneal fluorescein staining | OSDI |
| Kim et al. 2012 | 150 | 2 | Medical device therapy | Active | N/A | N/A | TFBUT | OSDI |
| Kim et al. 2019 | 54 | 2 | Nutritional supplements | Active | N/A | N/A | TFBUT | OSDI |
| Kim et al. 2017 | 144 | 3 | Topical medication | Active | Active | N/A | TFBUT | OSDI |
| Kinoshita et al. 2012 | 308 | 3 | Topical medication | Active | Placebo | N/A | Corneal fluorescein staining | Other |
| Kinoshita et al. 2013 | 188 | 2 | Topical medication | Active | N/A | N/A | Corneal fluorescein staining | Other |
| Kumar et al. 2021 | 56 | 2 | Topical lubricant | Active | N/A | N/A | TFBUT | OSDI |
| Kumari et al. 2023 | 44 | 2 | Topical medication | Active | N/A | N/A | Not defined | OSDI |
| Labetoulle et al. 2017 | 94 | 2 | Topical lubricant | Active | N/A | N/A | Ocular surface staining | IDEEL |
| Labetoulle et al. 2017 | 80 | 2 | Topical lubricant | Active | N/A | N/A | Ocular surface staining | OSDI |
| Labetoulle and Mortemousque 2022 | 83 | 2 | Topical lubricant | Active | N/A | N/A | Ocular surface staining | Other |
| Labetoulle et al. 2018 | 99 | 2 | Topical lubricant | Active | N/A | N/A | Ocular surface staining | IDEEL |
| Larmo et al. 2010 | 100 | 2 | Nutritional supplements | Placebo | N/A | N/A | Tear film osmolarity | OSDI |
| Lee et al. 2014 | 95 | 2 | Medical device therapy | Active | N/A | N/A | TFBUT | OSDI |
| Lee et al. 2022 | 364 | 3 | Topical lubricant | Active | Active | N/A | Corneal fluorescein staining | OSDI |
| Lee et al. 2011 | 67 | 2 | Topical lubricant | Active | N/A | N/A | Corneal fluorescein staining | Other |
| Lee et al. 2017 | 100 | 2 | Medical device therapy | Placebo | N/A | N/A | TFBUT | Other |
| Leonardi et al. 2016 | 261 | 2 | Topical medication | Placebo | N/A | N/A | Corneal fluorescein staining | OSDI |
| Lievens et al. 2019 | 188 | 2 | Topical lubricant | Active | N/A | N/A | Not defined | OSDI |
| Liew et al. 2012 | 327 | 3 | Topical medication | Active | Placebo | N/A | Schirmer I | Other |
| Lin et al. 2021 | 50 | 2 | Systemic medication | Active | N/A | N/A | TFBUT | OSDI |
| Liu et al. 2012 | 60 | 2 | Topical medication | Active | N/A | N/A | TFBUT | OSDI |
| Matsumoto et al. 2012 | 286 | 3 | Topical medication | Active | Placebo | N/A | Corneal fluorescein staining | Other |
| McCann et al. 2012 | 75 | 3 | Topical lubricant | Active | Active | N/A | Other | SANDE |
| Meerovitch et al. 2013 | 150 | 3 | Topical medication | Active | Placebo | N/A | Corneal fluorescein staining | Other |
| Mun et al. 2019 | 47 | 2 | Topical medication | Placebo | N/A | N/A | Ocular surface staining | OSDI |
| Muñoz-Villegas et al. 2023 | 116 | 3 | Topical lubricant | Active | Active | N/A | TFBUT | OSDI |
| Nava-Castaneda et al. 2003 | 61 | 2 | Medical device therapy | Placebo | N/A | N/A | Not defined | Other |
| Ng et al. 2022 | 60 | 2 | Nutritional supplements | Placebo | N/A | N/A | Schirmer I | OSDI |
| Ng et al. 2022 | 104 | 4 | Nutritional supplements | Active | Placebo | Active + Placebo | NITFBUT | OSDI |
| Oydanich et al. 2020 | 535 | 2 | Nutritional supplements | Placebo | N/A | N/A | Not defined | OSDI |
| Papa et al. 2001 | 158 | 2 | Topical lubricant | Active | N/A | N/A | Ocular surface staining | VAS |
| Park et al. 2019 | 227 | 3 | Topical medication | Active | Active | N/A | Ocular surface staining | OSDI |
| Park et al. 2022 | 40 | 2 | Medical device therapy | Placebo | N/A | N/A | Corneal fluorescein staining | OSDI |
| Park et al. 2017 | 176 | 4 | Topical lubricant | Active | Active | Active | Corneal fluorescein staining | OSDI |
| Patane et al. 2011 | 105 | 3 | Topical medication | Active | Placebo | N/A | Corneal fluorescein staining | Other |
| Peng et al. 2022 | 644 | 2 | Topical medication | Placebo | N/A | N/A | Corneal fluorescein staining | EDS |
| Peng et al. 2021 | 240 | 4 | Topical medication | Active | Active | Active | TFBUT | VAS |
| Pérez-Balbuena et al. 2016 | 183 | 2 | Topical lubricant | Active | N/A | N/A | Schirmer II | OSDI |
| Petrov et al. 2016 | 91 | 3 | Topical medication | Active | Placebo | N/A | Corneal fluorescein staining | Other |
| Pinto-Fraga et al. 2016 | 41 | 2 | Topical medication | Active | N/A | N/A | Corneal fluorescein staining | SANDE |
| Piwkumsribonruang et al. 2010 | 42 | 2 | Systemic medication | Placebo | N/A | N/A | Schirmer I | VAS |
| Postorino et al. 2018 | 40 | 2 | Topical medication | Active | N/A | N/A | Ocular surface staining | OSDI |
| Qin et al. 2023 | 60 | 2 | Medical device therapy | Placebo | N/A | N/A | NITFBUT | OSDI |
| Quiroz-Mercado et al. 2022 | 123 | 3 | Systemic medication | Active | Placebo | N/A | Schirmer II | Not defined |
| Radkar et al. 2021 | 60 | 2 | Nutritional supplements | Placebo | N/A | N/A | Schirmer I | OSDI |
| Rajendraprasad et al. 2021 | 180 | 2 | Topical lubricant | Active | N/A | N/A | TFBUT | OSDI |
| Rajpoot et al. 2022 | 467 | 2 | Topical medication | Active | N/A | N/A | Tear film osmolarity | Other |
| Rao 2010 | 58 | 2 | Topical medication | Active | N/A | N/A | Other | OSDI |
| Ren et al. 2020 | 76 | 4 | Nutritional supplements | Active | Active | Active | Other | Other |
| Ren et al. 2022 | 199 | 2 | Nutritional supplements | Active | N/A | N/A | Other | OSDI |
| Robert et al. 2016 | 85 | 2 | Topical lubricant | Active | N/A | N/A | Ocular surface staining | VAS |
| Rodríguez Calvo-de-Mora et al. 2022 | 63 | 3 | Topical medication | Active | Active | N/A | Schirmer I | Other |
| Rolando et al. 2023 | 40 | 2 | Topical medication | Active | N/A | N/A | Corneal fluorescein staining | SANDE |
| Safarzadeh et al. 2017 | 88 | 2 | Topical lubricant | Active | N/A | N/A | TFBUT | OSDI |
| Saffar Shahroodi et al. 2019 | 105 | 3 | Nutritional supplements | Placebo | Placebo | N/A | Schirmer I | OSDI |
| Salim et al. 2023 | 60 | 2 | Topical lubricant | Active | N/A | N/A | TFBUT | OSDI |
| Sall et al. 2000 | 877 | 3 | Topical medication | Active | Placebo | N/A | Corneal fluorescein staining | OSDI |
| Sánchez-González et al. 2023 | 60 | 2 | Topical lubricant | Active | N/A | N/A | Not defined | SPEED |
| Saubhagya Sindhu 2015 | 60 | 2 | Topical medication | Active | N/A | N/A | Schirmer I | OSDI |
| Schechter 2006 | 52 | 2 | Topical medication | Active | N/A | N/A | Corneal fluorescein staining | Other |
| Schechter et al. 2022 | 455 | 3 | Topical medication | Active | Placebo | N/A | Ocular surface staining | SANDE |
| Schmidl et al. 2020 | 48 | 2 | Topical lubricant | Placebo | N/A | N/A | Other | Not defined |
| Schmidl et al. 2021 | 104 | 4 | Topical medication | Active+Placebo | Active | Placebo | Schirmer II | Not defined |
| Scuderi et al. 2012 | 66 | 2 | Systemic medication | Placebo | N/A | N/A | Not defined | OSDI |
| Semba et al. 2012 | 230 | 4 | Topical medication | Active | Active | Placebo | Corneal fluorescein staining | OSDI |
| Serrano-Morales et al. 2022 | 60 | 2 | Topical lubricant | Active | N/A | N/A | Schirmer I | OSDI |
| Shah et al. 2017 | 90 | 2 | Topical medication | Active | N/A | N/A | Other | VAS |
| Shemer et al. 2023 | 40 | 2 | Medical device therapy | Placebo | N/A | N/A | Other | OSDI |
| Sheppard et al. 2014 | 588 | 2 | Topical medication | Placebo | N/A | N/A | Corneal fluorescein staining | Other |
| Sheppard et al. 2021 | 328 | 2 | Topical medication | Placebo | N/A | N/A | Corneal fluorescein staining | OSDI |
| Shettle et al. 2022 | 134 | 2 | Topical medication | Placebo | N/A | N/A | Not defined | Other |
| Shin et al. 2021 | 114 | 4 | Topical medication | Active | Active | Placebo | Corneal fluorescein staining | SPEED |
| Shin et al. 2010 | 42 | 2 | Medical device therapy | Placebo | N/A | N/A | TFBUT | OSDI |
| Simmons et al. 2015 | 315 | 4 | Topical lubricant | Active | Active | Active | TFBUT | OSDI |
| Simmons et al. 2015 | 288 | 3 | Topical lubricant | Active | Active | N/A | TFBUT | Other |
| Simmons et al. 2015 | 305 | 3 | Topical lubricant | Active | Active | N/A | Not defined | OSDI |
| Singh 2022 | 100 | 2 | Topical medication | Active | N/A | N/A | Ocular surface staining | OSDI |
| Singla et al. 2019 | 140 | 2 | Topical medication | Active | N/A | N/A | TFBUT | OSDI |
| Song et al. 2022 | 106 | 2 | Medical device therapy | Placebo | N/A | N/A | Other | OSDI |
| Sosne and Ousler 2015 | 72 | 2 | Topical medication | Placebo | N/A | N/A | Corneal fluorescein staining | Other |
| Stevenson et al. 2000 | 162 | 2 | Topical medication | Placebo | N/A | N/A | Ocular surface staining | Other |
| Su et al. 2011 | 100 | 2 | Topical medication | Active | N/A | N/A | TFBUT | OSDI |
| Supalaset et al. 2019 | 50 | 2 | Systemic medication | Placebo | N/A | N/A | Not defined | OSDI |
| Takamura et al. 2012 | 287 | 2 | Topical medication | Active | N/A | N/A | Corneal fluorescein staining | Other |
| Tan et al. 2020 | 46 | 2 | Topical lubricant | Active | N/A | N/A | Other | OSDI |
| Tauber et al. 2004 | 527 | 3 | Topical medication | Active | Placebo | N/A | Corneal fluorescein staining | Other |
| Tauber et al. 2023 | 170 | 3 | Topical medication | Active | Placebo | N/A | Corneal fluorescein staining | VAS |
| Tauber et al. 2015 | 718 | 2 | Topical medication | Placebo | N/A | N/A | Corneal fluorescein staining | VAS |
| Tauber et al. 2018 | 455 | 3 | Topical medication | Active | Placebo | N/A | Ocular surface staining | Other |
| Taylor et al. 2019 | 61 | 2 | Topical medication | Placebo | N/A | N/A | Ocular surface staining | Other |
| Titiyal et al. 2019 | 150 | 2 | Topical medication | Active | N/A | N/A | Other | OSDI |
| Tong et al. 2018 | 150 | 3 | Medical device therapy | Active | Active | N/A | Not defined | SPEED |
| Torkildsen et al. 2022 | 165 | 4 | Systemic medication | Active | Active | Placebo | Schirmer II | EDS |
| Toshida et al. 2017 | 66 | 2 | Topical medication | Placebo | N/A | N/A | Corneal fluorescein staining | Other |
| Utsunomiya et al. 2017 | 63 | 2 | Topical medication | Active | N/A | N/A | Corneal fluorescein staining | Other |
| van Setten et al. 2020 | 140 | 2 | Topical lubricant | Active | N/A | N/A | Corneal fluorescein staining | OSDI |
| Villani et al. 2011 | 60 | 2 | Topical lubricant | Placebo | N/A | N/A | TFBUT | OSDI |
| Vogel et al. 2010 | 444 | 2 | Topical lubricant | Placebo | N/A | N/A | Ocular surface staining | Other |
| Wang et al. 2007 | 80 | 3 | Topical lubricant | Active | Active | N/A | Schirmer II | Other |
| Watts et al. 2020 | 90 | 3 | Topical medication | Active | Active | N/A | TFBUT | OSDI |
| Wirta et al. 2022 | 758 | 3 | Systemic medication | Active | Placebo | N/A | Schirmer II | EDS |
| Wirta et al. 2022 | 369 | 3 | Topical medication | Active | Placebo | N/A | Schirmer II | VAS |
| Wirta et al. 2022 | 182 | 4 | Systemic medication | Active | Active | Placebo | Schirmer II | EDS |
| Wirta et al. 2019 | 207 | 4 | Topical medication | Active | Placebo | Active | Corneal fluorescein staining | VAS |
| Wu et al. 2021 | 53 | 2 | Topical medication | Active | N/A | N/A | Corneal fluorescein staining | OSDI |
| Yang et al. 2023 | 84 | 2 | Medical device therapy | Active | N/A | N/A | NITFBUT | OSDI |
| Yang et al. 2015 | 60 | 2 | Topical medication | Active | N/A | N/A | TFBUT | OSDI |
| Yang and Wang 2021 | 72 | 2 | Topical medication | Active | N/A | N/A | Schirmer I | Other |
| Zheng and Zhu 2023 | 240 | 2 | Topical medication | Active | N/A | N/A | TFBUT | OSDI |

*Supplementary Table 1: Listing of all included studies and their extracted variables. Abbreviations: TFBUT (Tear Film Break Up Time), NITFBUT (Non Invasive Tear Film Break Up Time), OSDI (Ocular Surface Disease Index), VAS (Visual Analogue Scale), IDEEL (Impact of Dry Eye on Everyday Life), SPEED (Standard Patient Evaluation of Eye Dryness Questionnaire), EDS (Eye Dryness Score), SANDE (Symptom Assessment Questionnaire in Dry Eye*

Aguilar A J, Marquez M I, Albera P A, Tredicce J L and Berra A (2014). Effects of Systane(®) Balance on noninvasive tear film break-up time in patients with lipid-deficient dry eye. Clin Ophthalmol **8**: 2365-2372.

Akpek E K, Wirta D L, Downing J E, Tauber J, Sheppard J D, Ciolino J B, . . . Krösser S (2023). Efficacy and Safety of a Water-Free Topical Cyclosporine, 0.1%, Solution for the Treatment of Moderate to Severe Dry Eye Disease: The ESSENCE-2 Randomized Clinical Trial. JAMA OPHTHALMOLOGY **141**(5): 459-466.

Aragona P, Benítez-Del-Castillo J M, Coroneo M T, Mukherji S, Tan J, Vandewalle E, . . . Simmons P A (2020). Safety and Efficacy of a Preservative-Free Artificial Tear Containing Carboxymethylcellulose and Hyaluronic Acid for Dry Eye Disease: A Randomized, Controlled, Multicenter 3-Month Study. Clin Ophthalmol **14**: 2951-2963.

Aragona P, Papa V, Micali A, Santocono M and Milazzo G (2002). Long term treatment with sodium hyaluronate-containing artificial tears reduces ocular surface damage in patients with dry eye. Br J Ophthalmol **86**(2): 181-184.

Asbell P, Vingrys A J, Tan J, Ogundele A, Downie L E, Jerkins G, . . . Shettle L (2018). Clinical Outcomes of Fixed Versus As-Needed Use of Artificial Tears in Dry Eye Disease: A 6-Week, Observer-Masked Phase 4 Clinical Trial. Invest Ophthalmol Vis Sci **59**(6): 2275-2280.

Asbell P A, Maguire M G, Pistilli M, Ying G S, Szczotka-Flynn L B, Hardten D R, . . . Shtein R M (2018). n-3 Fatty Acid Supplementation for the Treatment of Dry Eye Disease. N Engl J Med **378**(18): 1681-1690.

Avni I, Garzozi H J, Barequet I S, Segev F, Varssano D, Sartani G, . . . Fishman P (2010). Treatment of dry eye syndrome with orally administered CF101: data from a phase 2 clinical trial. Ophthalmology **117**(7): 1287-1293.

Ayres B D, Bloomenstein M R, Loh J, Chester T, Saenz B, Echegoyen J, . . . Dickerson J E, Jr. (2023). A Randomized, Controlled Trial Comparing Tearcare(®) and Cyclosporine Ophthalmic Emulsion for the Treatment of Dry Eye Disease (SAHARA). Clin Ophthalmol **17**: 3925-3940.

Baiza-Durán L, Medrano-Palafox J, Hernández-Quintela E, Lozano-Alcazar J and Alaníz-de la O J (2010). A comparative clinical trial of the efficacy of two different aqueous solutions of cyclosporine for the treatment of moderate-to-severe dry eye syndrome. Br J Ophthalmol **94**(10): 1312-1315.

Baiza-Durán L M, Muñoz-Villegas P, Sánchez-Ríos A and Olvera-Montaño O (2023). Efficacy and Safety of an Ophthalmic DMPC-Based Nanoemulsion in Patients with Dry Eye Disease: A Phase I/II Randomized Clinical Trial. J Ophthalmol **2023**: 1431473.

Barabino S, Rolando M, Nardi M, Bonini S, Aragona P and Traverso C E (2014). The effect of an artificial tear combining hyaluronic acid and tamarind seeds polysaccharide in patients with moderate dry eye syndrome: a new treatment for dry eye. Eur J Ophthalmol **24**(2): 173-178.

Baudouin C, Cochener B, Pisella P J, Girard B, Pouliquen P, Cooper H, . . . Creuzot-Garcher C (2012). Randomized, phase III study comparing osmoprotective carboxymethylcellulose with sodium hyaluronate in dry eye disease. Eur J Ophthalmol **22**(5): 751-761.

Baudouin C, de la Maza M S, Amrane M, Garrigue J S, Ismail D, Figueiredo F C, . . . Leonardi A (2017). One-Year Efficacy and Safety of 0.1% Cyclosporine a Cationic Emulsion in the Treatment of Severe Dry Eye Disease. Eur J Ophthalmol **27**(6): 678-685.

Baudouin C, Figueiredo F C, Messmer E M, Ismail D, Amrane M, Garrigue J S, . . . Leonardi A (2017). A randomized study of the efficacy and safety of 0.1% cyclosporine A cationic emulsion in treatment of moderate to severe dry eye. Eur J Ophthalmol **27**(5): 520-530.

Belalcázar-Rey S, Sánchez Huerta V, Ochoa-Tabares J C, Altamirano Vallejo S, Soto-Gómez A, Suárez-Velasco R, . . . Muñoz-Villegas P (2021). Efficacy and Safety of Sodium Hyaluronate/chondroitin Sulfate Preservative-free Ophthalmic Solution in the Treatment of Dry Eye: A Clinical Trial. Curr Eye Res **46**(7): 919-929.

Benelli U, Nardi M, Posarelli C and Albert T G (2010). Tear osmolarity measurement using the TearLab Osmolarity System in the assessment of dry eye treatment effectiveness. Cont Lens Anterior Eye **33**(2): 61-67.

Bhargava R, Kumar P, Phogat H, Kaur A and Kumar M (2015). Oral omega-3 fatty acids treatment in computer vision syndrome related dry eye. Cont Lens Anterior Eye **38**(3): 206-210.

Bhargava R, Pandey K, Ranjan S, Mehta B and Malik A (2023). Omega-3 fatty acids supplements for dry eye - Are they effective or ineffective? Indian J Ophthalmol **71**(4): 1619-1625.

Bing Zhang P-W Z, Qi Lin, Qing Yuan, Biao Li, You-Lan Min, Lei Ye, Wen-Qing Shi, Yi Shao (2019 ). Buddleja officinalis eye drops alleviate the symptoms of moderate to severe dry eye. International Journal of Clinical and Experimental Medicine **12**(7): 9277-9287.

Brar S, Vanga H R and Ganesh S (2021). Comparison of efficacy of trehalose-based eye drops versus topical 0.1% Hyaluronic Acid for management of clinically significant dry eye using non-invasive investigational modalities. Int Ophthalmol **41**(10): 3349-3359.

Brignole-Baudouin F, Baudouin C, Aragona P, Rolando M, Labetoulle M, Pisella P J, . . . Creuzot-Garcher C (2011). A multicentre, double-masked, randomized, controlled trial assessing the effect of oral supplementation of omega-3 and omega-6 fatty acids on a conjunctival inflammatory marker in dry eye patients. Acta Ophthalmol **89**(7): e591-597.

Brignole F, Pisella P J, De Saint Jean M, Goldschild M, Goguel A and Baudouin C (2001). Flow cytometric analysis of inflammatory markers in KCS: 6-month treatment with topical cyclosporin A. Invest Ophthalmol Vis Sci **42**(1): 90-95.

Brzheskiy V V, Efimova E L, Vorontsova T N, Alekseev V N, Gusarevich O G, Shaidurova K N, . . . Skulachev M V (2015). Results of a Multicenter, Randomized, Double-Masked, Placebo-Controlled Clinical Study of the Efficacy and Safety of Visomitin Eye Drops in Patients with Dry Eye Syndrome. Adv Ther **32**(12): 1263-1279.

Byun Y J, Kim T I, Kwon S M, Seo K Y, Kim S W, Kim E K, . . . Park W C (2012). Efficacy of combined 0.05% cyclosporine and 1% methylprednisolone treatment for chronic dry eye. Cornea **31**(5): 509-513.

Cai M M and Zhang J (2020). Effectiveness of transcutaneous electrical stimulation combined with artificial tears for the treatment of dry eye: A randomized controlled trial. Exp Ther Med **20**(6): 175.

Calonge M, Sahyoun M, Baillif S, Gain P, Paw E, Mearza A, . . . Cochener B (2023). Sodium hyaluronate 0.30% ocular gel versus sodium hyaluronate 0.18% eye drop in the treatment of moderate to severe dry eye disease. Eur J Ophthalmol **33**(1): 188-195.

Carreira A R, Rodrigues-Barros S, Silva J C, de Almeida M F, Machado I, Cardoso J N, . . . Campos N (2023). Tobacco effects on ocular surface, meibomian glands, and corneal epithelium and the benefits of treatment with a lipid-based lubricant. Graefes Arch Clin Exp Ophthalmol **261**(1): 171-184.

Chen D, Zhang S, Bian A, Hong J, Deng Y, Zhang M, . . . Zhao J (2019). Efficacy and safety of 0.05% cyclosporine ophthalmic emulsion in treatment of Chinese patients with moderate to severe dry eye disease: A 12-week, multicenter, randomized, double-masked, placebo-controlled phase III clinical study. Medicine (Baltimore) **98**(31): e16710.

Chen J, Dong F, Chen W, Sun X, Deng Y, Hong J, . . . Xie L (2014). Clinical efficacy of 0.1% pranoprofen in treatment of dry eye patients: a multicenter, randomized, controlled clinical trial. Chin Med J (Engl) **127**(13): 2407-2412.

Chen J, Qin G, Li L, Qi Y, Xia Y, Zhang Q, . . . He X (2023). The Combined Impact of Intense Pulsed Light Combined and 3% Diquafosol Ophthalmic Solution on Evaporative Dry Eye: A Randomized Control Study. Ophthalmol Ther **12**(6): 2959-2971.

Chen M, Gong L, Sun X, Xie H, Zhang Y, Zou L, . . . He J (2010). A comparison of cyclosporine 0.05% ophthalmic emulsion versus vehicle in Chinese patients with moderate to severe dry eye disease: an eight-week, multicenter, randomized, double-blind, parallel-group trial. J Ocul Pharmacol Ther **26**(4): 361-366.

Chiambaretta F, Doan S, Labetoulle M, Rocher N, Fekih L E, Messaoud R, . . . Baudouin C (2017). A randomized, controlled study of the efficacy and safety of a new eyedrop formulation for moderate to severe dry eye syndrome. Eur J Ophthalmol **27**(1): 1-9.

Cho Y K, Huang W, Kim G Y and Lim B S (2013). Comparison of autologous serum eye drops with different diluents. Curr Eye Res **38**(1): 9-17.

Choi W, Kim J C, Kim W S, Oh H J, Yang J M, Lee J B, . . . Yoon K C (2015). Clinical Effect of Antioxidant Glasses Containing Extracts of Medicinal Plants in Patients with Dry Eye Disease: A Multi-Center, Prospective, Randomized, Double-Blind, Placebo-Controlled Trial. PLoS One **10**(10): e0139761.

Christensen M T, Cohen S, Rinehart J, Akers F, Pemberton B, Bloomenstein M, . . . Stein J M (2004). Clinical evaluation of an HP-guar gellable lubricant eye drop for the relief of dryness of the eye. Curr Eye Res **28**(1): 55-62.

Chung S H, Lim S A and Tchach H (2016). Efficacy and Safety of Carbomer-Based Lipid-Containing Artificial Tear Formulations in Patients With Dry Eye Syndrome. Cornea **35**(2): 181-186.

Clark D, Tauber J, Sheppard J and Brady T C (2021). Early Onset and Broad Activity of Reproxalap in a Randomized, Double-Masked, Vehicle-Controlled Phase 2b Trial in Dry Eye Disease. Am J Ophthalmol **226**: 22-31.

Cohen S, Martin A and Sall K (2014). Evaluation of clinical outcomes in patients with dry eye disease using lubricant eye drops containing polyethylene glycol or carboxymethylcellulose. Clin Ophthalmol **8**: 157-164.

Cohn G S, Corbett D, Tenen A, Coroneo M, McAlister J, Craig J P, . . . Holland E J (2019). Randomized, Controlled, Double-Masked, Multicenter, Pilot Study Evaluating Safety and Efficacy of Intranasal Neurostimulation for Dry Eye Disease. Invest Ophthalmol Vis Sci **60**(1): 147-153.

Craig J P, Muntz A, Wang M T M, Luensmann D, Tan J, Trave Huarte S, . . . Wolffsohn J S (2021). Developing evidence-based guidance for the treatment of dry eye disease with artificial tear supplements: A six-month multicentre, double-masked randomised controlled trial. Ocul Surf **20**: 62-69.

Davitt W F, Bloomenstein M, Christensen M and Martin A E (2010). Efficacy in patients with dry eye after treatment with a new lubricant eye drop formulation. J Ocul Pharmacol Ther **26**(4): 347-353.

Deinema L A, Vingrys A J, Wong C Y, Jackson D C, Chinnery H R and Downie L E (2017). A Randomized, Double-Masked, Placebo-Controlled Clinical Trial of Two Forms of Omega-3 Supplements for Treating Dry Eye Disease. Ophthalmology **124**(1): 43-52.

Dong Y, Wang S, Cong L, Zhang T, Cheng J, Yang N, . . . Xie L (2022). TNF-α inhibitor tanfanercept (HBM9036) improves signs and symptoms of dry eye in a phase 2 trial in the controlled adverse environment in China. Int Ophthalmol **42**(8): 2459-2472.

Downie L E, Hom M M, Berdy G J, El-Harazi S, Verachtert A, Tan J, . . . Vehige J (2020). An artificial tear containing flaxseed oil for treating dry eye disease: A randomized controlled trial. Ocul Surf **18**(1): 148-157.

Eom Y, Chung S H, Chung T-Y, Kim J Y, Choi C Y, Yoon K C, . . . Kim H M (2023). Efficacy and safety of 1% and 2% rebamipide clear solution in dry eye disease: a multicenter randomized trial. BMC OPHTHALMOLOGY **23**(1): 343.

Eom Y and Kim H M (2021). Clinical effectiveness of diquafosol ophthalmic solution 3% in Korean patients with dry eye disease: a multicenter prospective observational study. Int J Ophthalmol **14**(10): 1518-1526.

Eom Y, Yoon K C, Kim H K, Song J S, Hyon J Y and Kim H M (2023). A Multicenter, Randomized, Double-Blind Evaluation of the Efficacy of TJO-087 Versus 0.05% Cyclosporine A in Moderate to Severe Dry Eye. J Ocul Pharmacol Ther **39**(1): 27-35.

Epitropoulos A T, Donnenfeld E D, Shah Z A, Holland E J, Gross M, Faulkner W J, . . . Perry H D (2016). Effect of Oral Re-esterified Omega-3 Nutritional Supplementation on Dry Eyes. Cornea **35**(9): 1185-1191.

Essa L, Laughton D and Wolffsohn J S (2018). Can the optimum artificial tear treatment for dry eye disease be predicted from presenting signs and symptoms? Cont Lens Anterior Eye **41**(1): 60-68.

Evans D, Kenyon K, Ousler G, Watson M, Vollmer P, McLaurin E B, . . . Spana C (2023). Efficacy and Safety of the Melanocortin Pan-Agonist PL9643 in a Phase 2 Study of Patients with Dry Eye Disease. J Ocul Pharmacol Ther **39**(9): 600-610.

Fan M, Kim S A, Choi Y J, Tang Y, Yang H P and Kim E K (2023). Anthocyanin oligomer (grape skin extract) administration improves dry eye disease: A randomised, double-blind, placebo-controlled study. Clin Exp Ophthalmol **51**(2): 122-130.

Fondi K, Wozniak P A, Schmidl D, Bata A M, Witkowska K J, Popa-Cherecheanu A, . . . Garhöfer G (2018). Effect of Hyaluronic Acid/Trehalose in Two Different Formulations on Signs and Symptoms in Patients with Moderate to Severe Dry Eye Disease. J Ophthalmol **2018**: 4691417.

García-Conca V, Abad-Collado M, Hueso-Abancens J R, Mengual-Verdú E, Piñero D P, Aguirre-Balsalobre F, . . . Molina J C (2019). Efficacy and safety of treatment of hyposecretory dry eye with platelet-rich plasma. Acta Ophthalmol **97**(2): e170-e178.

Goldberg D F, Malhotra R P, Schechter B A, Justice A, Weiss S L and Sheppard J D (2019). A Phase 3, Randomized, Double-Masked Study of OTX-101 Ophthalmic Solution 0.09% in the Treatment of Dry Eye Disease. Ophthalmology **126**(9): 1230-1237.

Goldstein M H, Martel J R, Sall K, Goldberg D F, Abrams M, Rubin J, . . . Furfine E (2017). Multicenter Study of a Novel Topical Interleukin-1 Receptor Inhibitor, Isunakinra, in Subjects With Moderate to Severe Dry Eye Disease. Eye Contact Lens **43**(5): 287-296.

Gong L, Sun X, Ma Z, Wang Q, Xu X, Chen X, . . . Tong L (2015). A randomised, parallel-group comparison study of diquafosol ophthalmic solution in patients with dry eye in China and Singapore. Br J Ophthalmol **99**(7): 903-908.

Groß D, Childs M and Piaton J M (2017). Comparison of 0.2% and 0.18% hyaluronate eye drops in patients with moderate to severe dry eye with keratitis or keratoconjunctivitis. Clin Ophthalmol **11**: 631-638.

Groß D, Childs M and Piaton J M (2018). Comparative study of 0.1% hyaluronic acid versus 0.5% carboxymethylcellulose in patients with dry eye associated with moderate keratitis or keratoconjunctivitis. Clin Ophthalmol **12**: 1081-1088.

Grosskreutz C L, Hockey H U, Serra D and Dryja T P (2015). Dry Eye Signs and Symptoms Persist During Systemic Neutralization of IL-1β by Canakinumab or IL-17A by Secukinumab. Cornea **34**(12): 1551-1556.

Haji-Ali-Nili N, Khoshzaban F, Karimi M, Roja R, Ashrafi E, Ghaffari R, . . . Jabarvand Behrouz M (2019). Effect of a Natural Eye Drop, Made of Plantago Ovata Mucilage on Improvement of Dry Eye Symptoms: A Randomized, Double-blind Clinical Trial. Iran J Pharm Res **18**(3): 1602-1611.

Hassan A, Balal S, Cook E, Dehbi H M, Pardhan S, Bourne R, . . . Sharma A (2022). Finger-Prick Autologous Blood (FAB) Eye Drops for Dry Eye Disease: Single Masked Multi-Centre Randomised Controlled Trial. Clin Ophthalmol **16**: 3973-3979.

Holland E J, Luchs J, Karpecki P M, Nichols K K, Jackson M A, Sall K, . . . Shojaei A (2017). Lifitegrast for the Treatment of Dry Eye Disease: Results of a Phase III, Randomized, Double-Masked, Placebo-Controlled Trial (OPUS-3). Ophthalmology **124**(1): 53-60.

Hori Y, Oka K and Inai M (2022). Efficacy and Safety of the Long-Acting Diquafosol Ophthalmic Solution DE-089C in Patients with Dry Eye: A Randomized, Double-Masked, Placebo-Controlled Phase 3 Study. Adv Ther **39**(8): 3654-3667.

Hovanesian J A, Keyser A, Berdy G and Sorensen R (2022). The DEPOT Study (Dry Eye Prescription Options for Therapy): Assessing the Efficacy and Safety of OTX-DED (Dexamethasone Ophthalmic Insert 0.3 mg) for Intracanalicular Use Compared with Loteprednol Suspension for the Treatment of Episodic Dry Eye. Clin Ophthalmol **16**: 3841-3849.

Hu W L, Yu H J, Pan L Y, Wu P C, Pan C C, Kuo C E, . . . Hung Y C (2021). Laser Acupuncture Improves Tear Film Stability in Patients with Dry Eye Disease: A Two-Center Randomized-Controlled Trial. J Altern Complement Med **27**(7): 579-587.

Huang J Y, Yeh P T and Hou Y C (2016). A randomized, double-blind, placebo-controlled study of oral antioxidant supplement therapy in patients with dry eye syndrome. Clin Ophthalmol **10**: 813-820.

Hussain M, Shtein R M, Pistilli M, Maguire M G, Oydanich M and Asbell P A (2020). The Dry Eye Assessment and Management (DREAM) extension study - A randomized clinical trial of withdrawal of supplementation with omega-3 fatty acid in patients with dry eye disease. Ocul Surf **18**(1): 47-55.

Hwang H S, Sung Y M, Lee W S and Kim E C (2014). Additive Effect of preservative-free sodium hyaluronate 0.1% in treatment of dry eye syndrome with diquafosol 3% eye drops. Cornea **33**(9): 935-941.

Iester M, Orsoni G J, Gamba G, Taffara M, Mangiafico P, Giuffrida S, . . . Rolando M (2000). Improvement of the ocular surface using hypotonic 0.4% hyaluronic acid drops in keratoconjunctivitis sicca. Eye (Lond) **14**(Pt 6): 892-898.

Jackson M A, Burrell K, Gaddie I B and Richardson S D (2011). Efficacy of a new prescription-only medical food supplement in alleviating signs and symptoms of dry eye, with or without concomitant cyclosporine A. Clin Ophthalmol **5**: 1201-1206.

Järvinen R L, Larmo P S, Setälä N L, Yang B, Engblom J R, Viitanen M H, . . . Kallio H P (2011). Effects of oral sea buckthorn oil on tear film Fatty acids in individuals with dry eye. Cornea **30**(9): 1013-1019.

Jee D, Park S H, Kim M S and Kim E C (2014). Antioxidant and inflammatory cytokine in tears of patients with dry eye syndrome treated with preservative-free versus preserved eye drops. Invest Ophthalmol Vis Sci **55**(8): 5081-5089.

Jerkins G, Greiner J V, Tong L, Tan J, Tauber J, Mearza A, . . . Srinivasan S (2020). A Comparison of Efficacy and Safety of Two Lipid-Based Lubricant Eye Drops for the Management of Evaporative Dry Eye Disease. Clin Ophthalmol **14**: 1665-1673.

Jiang N, Ye L H, Ye L, Yu J, Yang Q C, Yuan Q, . . . Shao Y (2017). Effect of mistletoe combined with carboxymethyl cellulose on dry eye in postmenopausal women. Int J Ophthalmol **10**(11): 1669-1677.

Jing Yu X-L Z, Yun-Yan Li, Pei-Hong Hu, Rong-Qiong Liu, Nan Jiang, Chong-Gang Pei, Yi Shao (2016). Clinical findings associated with Bidens bipinnata L. eye drops on moderate and severe dry eye in postmenopausal women. International Journal of Clinical and Experimental Medicine **9**(3): 3):5643-5656.

Jun J H, Bang S P, Park H S, Yoon D, Ahn J Y, Kim S J, . . . Kim H K (2022). A randomized multicenter clinical evaluation of sequential application of 0.3% and 0.15% hyaluronic acid for treatment of dry eye. Jpn J Ophthalmol **66**(1): 58-67.

Jung G T, Kim M, Song J S, Kim T I, Chung T Y, Choi C Y, . . . Lee H K (2023). Proteomic analysis of tears in dry eye disease: A prospective, double-blind multicenter study. Ocul Surf **29**: 68-76.

Kaercher T, Messmer E M, Berninger T, Huber-van der Velden K K, Geiger R, Cipriano-Bonvin P, . . . Jacobi C (2022). Topical Omega-3 Polyunsaturated Fatty Acids for the Treatment of Dry Eye - Results from a Pilot Randomized Controlled Masked-Observer Study. Clin Ophthalmol **16**: 4021-4031.

Kaido M, Ishida R, Dogru M and Tsubota K (2012). Visual function changes after punctal occlusion with the treatment of short BUT type of dry eye. Cornea **31**(9): 1009-1013.

Kallab M, Szegedi S, Hommer N, Stegmann H, Kaya S, Werkmeister R M, . . . Garhöfer G (2020). Topical Low Dose Preservative-Free Hydrocortisone Reduces Signs and Symptoms in Patients with Chronic Dry Eye: A Randomized Clinical Trial. Adv Ther **37**(1): 329-341.

Kan J, Wang M, Liu Y, Liu H, Chen L, Zhang X, . . . Du J (2020). A novel botanical formula improves eye fatigue and dry eye: a randomized, double-blind, placebo-controlled study. Am J Clin Nutr **112**(2): 334-342.

Kangari H, Eftekhari M H, Sardari S, Hashemi H, Salamzadeh J, Ghassemi-Broumand M, . . . Khabazkhoob M (2013). Short-term consumption of oral omega-3 and dry eye syndrome. Ophthalmology **120**(11): 2191-2196.

Kawakita T, Uchino M, Fukagawa K, Yoshino K, Shimazaki S, Toda I, . . . Tsubota K (2016). Randomized, Multicenter, Double-Blind Study of the Safety and Efficacy of 1%D-3-Hydroxybutyrate eye drops for Dry Eye Disease. Sci Rep **6**: 20855.

Kim E C, Choi J S and Joo C K (2009). A comparison of vitamin a and cyclosporine a 0.05% eye drops for treatment of dry eye syndrome. Am J Ophthalmol **147**(2): 206-213.e203.

Kim H S, Kim T I, Kim J H, Yoon K C, Hyon J Y, Shin K U, . . . Choi C Y (2017). Evaluation of Clinical Efficacy and Safety of a Novel Cyclosporin A Nanoemulsion in the Treatment of Dry Eye Syndrome. J Ocul Pharmacol Ther **33**(7): 530-538.

Kim T H, Kang J W, Kim K H, Kang K W, Shin M S, Jung S Y, . . . Choi S M (2012). Acupuncture for the treatment of dry eye: a multicenter randomised controlled trial with active comparison intervention (artificial teardrops). PLoS One **7**(5): e36638.

Kim Y, Moon C H, Kim B Y and Jang S Y (2019). Oral Hyaluronic Acid Supplementation for the Treatment of Dry Eye Disease: A Pilot Study. J Ophthalmol **2019**: 5491626.

Kim Y H, Kang Y S, Lee H S, Choi W, You I C and Yoon K C (2017). Effectiveness of Combined Tear Film Therapy in Patients with Evaporative Dry Eye with Short Tear Film Breakup Time. J Ocul Pharmacol Ther **33**(8): 635-643.

Kinoshita S, Awamura S, Oshiden K, Nakamichi N, Suzuki H and Yokoi N (2012). Rebamipide (OPC-12759) in the treatment of dry eye: a randomized, double-masked, multicenter, placebo-controlled phase II study. Ophthalmology **119**(12): 2471-2478.

Kinoshita S, Oshiden K, Awamura S, Suzuki H, Nakamichi N and Yokoi N (2013). A randomized, multicenter phase 3 study comparing 2% rebamipide (OPC-12759) with 0.1% sodium hyaluronate in the treatment of dry eye. Ophthalmology **120**(6): 1158-1165.

Kumar L, Kumar P and Jha A K (2021). A comparative study of preserved and preservative-free hydroxypropyl methylcellulose-dextran-containing eyedrops in dry eye disease. European Journal of Molecular and Clinical Medicine **8**: 1868+.

Kumari N, Kusumesh R, Kumari R, Sinha B P and Singh V (2023). Comparative evaluation of effectiveness of twenty versus fifty percent autologous serum eye drops in treatment of dry eye. Indian J Ophthalmol **71**(4): 1603-1607.

Labetoulle M, Chiambaretta F, Shirlaw A, Leaback R and Baudouin C (2017). Osmoprotectants, carboxymethylcellulose and hyaluronic acid multi-ingredient eye drop: a randomised controlled trial in moderate to severe dry eye. Eye (Lond) **31**(10): 1409-1416.

Labetoulle M, Messmer E M, Pisella P J, Ogundele A and Baudouin C (2017). Safety and efficacy of a hydroxypropyl guar/polyethylene glycol/propylene glycol-based lubricant eye-drop in patients with dry eye. Br J Ophthalmol **101**(4): 487-492.

Labetoulle M and Mortemousque B (2022). Performance and Safety of a Sodium Hyaluronate Tear Substitute with Polyethylene Glycol in Dry Eye Disease: A Multicenter, Investigator-Masked, Randomized, Noninferiority Trial. J Ocul Pharmacol Ther **38**(9): 607-616.

Labetoulle M, Schmickler S, Galarreta D, Böhringer D, Ogundele A, Guillon M, . . . Baudouin C (2018). Efficacy and safety of dual-polymer hydroxypropyl guar- and hyaluronic acid-containing lubricant eyedrops for the management of dry-eye disease: a randomized double-masked clinical study. Clin Ophthalmol **12**: 2499-2508.

Larmo P S, Järvinen R L, Setälä N L, Yang B, Viitanen M H, Engblom J R, . . . Kallio H P (2010). Oral sea buckthorn oil attenuates tear film osmolarity and symptoms in individuals with dry eye. J Nutr **140**(8): 1462-1468.

Lee J E, Kim N M, Yang J W, Kim S J, Lee J S and Lee J E (2014). A randomised controlled trial comparing a thermal massager with artificial teardrops for the treatment of dry eye. Br J Ophthalmol **98**(1): 46-51.

Lee J E, Kim S, Lee H K, Chung T Y, Kim J Y, Choi C Y, . . . Hyon J Y (2022). A randomized multicenter evaluation of the efficacy of 0.15% hyaluronic acid versus 0.05% cyclosporine A in dry eye syndrome. Sci Rep **12**(1): 18737.

Lee J H, Ahn H S, Kim E K and Kim T I (2011). Efficacy of sodium hyaluronate and carboxymethylcellulose in treating mild to moderate dry eye disease. Cornea **30**(2): 175-179.

Lee J S, Hwang S H, Shin B C and Park Y M (2017). Electrical stimulation of auricular acupressure for dry eye: A randomized controlled-clinical trial. Chin J Integr Med **23**(11): 822-828.

Leonardi A, Van Setten G, Amrane M, Ismail D, Garrigue J S, Figueiredo F C, . . . Baudouin C (2016). Efficacy and safety of 0.1% cyclosporine A cationic emulsion in the treatment of severe dry eye disease: a multicenter randomized trial. Eur J Ophthalmol **26**(4): 287-296.

Lievens C, Berdy G, Douglass D, Montaquila S, Lin H, Simmons P, . . . Haque S (2019). Evaluation of an enhanced viscosity artificial tear for moderate to severe dry eye disease: A multicenter, double-masked, randomized 30-day study. Cont Lens Anterior Eye **42**(4): 443-449.

Liew S H, Nichols K K, Klamerus K J, Li J Z, Zhang M and Foulks G N (2012). Tofacitinib (CP-690,550), a Janus kinase inhibitor for dry eye disease: results from a phase 1/2 trial. Ophthalmology **119**(7): 1328-1335.

Lin T, Wang W, Lu Y and Gong L (2021). Treatment of Dry Eye With Intracanalicular Injection of Hydroxybutyl Chitosan: A Prospective Randomized Clinical Trial. Front Med (Lausanne) **8**: 769448.

Liu X, Wang S, Kao A A and Long Q (2012). The effect of topical pranoprofen 0.1% on the clinical evaluation and conjunctival HLA-DR expression in dry eyes. Cornea **31**(11): 1235-1239.

Matsumoto Y, Ohashi Y, Watanabe H and Tsubota K (2012). Efficacy and safety of diquafosol ophthalmic solution in patients with dry eye syndrome: a Japanese phase 2 clinical trial. Ophthalmology **119**(10): 1954-1960.

McCann L C, Tomlinson A, Pearce E I and Papa V (2012). Effectiveness of artificial tears in the management of evaporative dry eye. Cornea **31**(1): 1-5.

Meerovitch K, Torkildsen G, Lonsdale J, Goldfarb H, Lama T, Cumberlidge G, . . . Ousler G W, 3rd (2013). Safety and efficacy of MIM-D3 ophthalmic solutions in a randomized, placebo-controlled Phase 2 clinical trial in patients with dry eye. Clin Ophthalmol **7**: 1275-1285.

Mun C, Gulati S, Tibrewal S, Chen Y F, An S, Surenkhuu B, . . . Jain S (2019). A Phase I/II Placebo-Controlled Randomized Pilot Clinical Trial of Recombinant Deoxyribonuclease (DNase) Eye Drops Use in Patients With Dry Eye Disease. Transl Vis Sci Technol **8**(3): 10.

Muñoz-Villegas P D C, Sánchez-Ríos A and Olvera-Montaño O (2023). The Effect of Sodium Hyaluronate Eye Drops 2, 4 or 6 Times a Day on Signs and Symptoms of Dry Eye Disease. Clin Ophthalmol **17**: 2945-2955.

Nava-Castaneda A, Tovilla-Canales J L, Rodriguez L, Tovilla Y P J L and Jones C E (2003). Effects of lacrimal occlusion with collagen and silicone plugs on patients with conjunctivitis associated with dry eye. Cornea **22**(1): 10-14.

Ng A, Woods J, Jahn T, Jones L W and Sullivan Ritter J (2022). Effect of a Novel Omega-3 and Omega-6 Fatty Acid Supplement on Dry Eye Disease: A 3-month Randomized Controlled Trial. Optom Vis Sci **99**(1): 67-75.

Ng D, Altamirano-Vallejo J C, Gonzalez-De la Rosa A, Navarro-Partida J, Valdez-Garcia J E, Acosta-Gonzalez R, . . . Santos A (2022). An Oral Polyphenol Formulation to Modulate the Ocular Surface Inflammatory Process and to Improve the Symptomatology Associated with Dry Eye Disease. NUTRIENTS **14**(15).

Oydanich M, Maguire M G, Pistilli M, Hamrah P, Greiner J V, Lin M C, . . . Asbell P A (2020). Effects of Omega-3 Supplementation on Exploratory Outcomes in the Dry Eye Assessment and Management Study. Ophthalmology **127**(1): 136-138.

Papa V, Aragona P, Russo S, Di Bella A, Russo P and Milazzo G (2001). Comparison of hypotonic and isotonic solutions containing sodium hyaluronate on the symptomatic treatment of dry eye patients. Ophthalmologica **215**(2): 124-127.

Park C H, Kim M K, Kim E C, Kim J Y, Kim T I, Kim H K, . . . Kim H S (2019). Efficacy of Topical Cyclosporine Nanoemulsion 0.05% Compared with Topical Cyclosporine Emulsion 0.05% and Diquafosol 3% in Dry Eye. Korean J Ophthalmol **33**(4): 343-352.

Park Y, Kim H, Kim S and Cho K J (2022). Effect of low-level light therapy in patients with dry eye: a prospective, randomized, observer-masked trial. Sci Rep **12**(1): 3575.

Park Y, Song J S, Choi C Y, Yoon K C, Lee H K and Kim H S (2017). A Randomized Multicenter Study Comparing 0.1%, 0.15%, and 0.3% Sodium Hyaluronate with 0.05% Cyclosporine in the Treatment of Dry Eye. J Ocul Pharmacol Ther **33**(2): 66-72.

Patane M A, Cohen A, From S, Torkildsen G, Welch D and Ousler G W, 3rd (2011). Ocular iontophoresis of EGP-437 (dexamethasone phosphate) in dry eye patients: results of a randomized clinical trial. Clin Ophthalmol **5**: 633-643.

Peng W, Jiang X, Zhu L, Li X, Zhou Q, Jie Y, . . . Zhou S (2022). Cyclosporine A (0.05%) Ophthalmic Gel in the Treatment of Dry Eye Disease: A Multicenter, Randomized, Double-Masked, Phase III, COSMO Trial. Drug Des Devel Ther **16**: 3183-3194.

Peng W Y, Chen R X, Dai H, Zhu L, Li Y, Gao Z Q, . . . Zhou S Y (2021). Efficacy, Safety, and Tolerability of a Novel Cyclosporine, a Formulation for Dry Eye Disease: A Multicenter Phase II Clinical Study. Clin Ther **43**(3): 613-628.

Pérez-Balbuena A L, Ochoa-Tabares J C, Belalcazar-Rey S, Urzúa-Salinas C, Saucedo-Rodríguez L R, Velasco-Ramos R, . . . Oregón-Miranda A A (2016). Efficacy of a fixed combination of 0.09 % xanthan gum/0.1 % chondroitin sulfate preservative free vs polyethylene glycol/propylene glycol in subjects with dry eye disease: a multicenter randomized controlled trial. BMC Ophthalmol **16**(1): 164.

Petrov A, Perekhvatova N, Skulachev M, Stein L and Ousler G (2016). SkQ1 Ophthalmic Solution for Dry Eye Treatment: Results of a Phase 2 Safety and Efficacy Clinical Study in the Environment and During Challenge in the Controlled Adverse Environment Model. Adv Ther **33**(1): 96-115.

Pinto-Fraga J, López-Miguel A, González-García M J, Fernández I, López-de-la-Rosa A, Enríquez-de-Salamanca A, . . . Calonge M (2016). Topical Fluorometholone Protects the Ocular Surface of Dry Eye Patients from Desiccating Stress: A Randomized Controlled Clinical Trial. Ophthalmology **123**(1): 141-153.

Piwkumsribonruang N, Somboonporn W, Luanratanakorn P, Kaewrudee S, Tharnprisan P and Soontrapa S (2010). Effectiveness of hormone therapy for treating dry eye syndrome in postmenopausal women: a randomized trial. J Med Assoc Thai **93**(6): 647-652.

Postorino E I, Rania L, Aragona E, Mannucci C, Alibrandi A, Calapai G, . . . Aragona P (2018). Efficacy of eyedrops containing cross-linked hyaluronic acid and coenzyme Q10 in treating patients with mild to moderate dry eye. Eur J Ophthalmol **28**(1): 25-31.

Qin G, Chen J, Li L, Xia Y, Zhang Q, Wu Y, . . . Pazo E E (2023). Managing Severe Evaporative Dry Eye with Intense Pulsed Light Therapy. Ophthalmol Ther **12**(2): 1059-1071.

Quiroz-Mercado H, Hernandez-Quintela E, Chiu K H, Henry E and Nau J A (2022). A phase II randomized trial to evaluate the long-term (12-week) efficacy and safety of OC-01 (varenicline solution) nasal spray for dry eye disease: The MYSTIC study. Ocul Surf **24**: 15-21.

Radkar P, Lakshmanan P S, Mary J J, Chaudhary S and Durairaj S K (2021). A Novel Multi-Ingredient Supplement Reduces Inflammation of the Eye and Improves Production and Quality of Tears in Humans. Ophthalmol Ther **10**(3): 581-599.

Rajendraprasad R M, Kwatra G and Batra N (2021). Carboxymethyl Cellulose versus Hydroxypropyl Methylcellulose Tear Substitutes for Dry Eye Due to Computer Vision Syndrome: Comparison of Efficacy and Safety. Int J Appl Basic Med Res **11**(1): 4-8.

Rajpoot M, Singh D, Pandey K and Bhargava R (2022). Safety and efficacy of cyclosporine (0.05% versus 0.09%) in dry eye disease. Is it the strength of cyclosporin that really matters? Nepal J Ophthalmol **14**(28): 64-77.

Rao S N (2010). Topical cyclosporine 0.05% for the prevention of dry eye disease progression. J Ocul Pharmacol Ther **26**(2): 157-164.

Ren X, Chou Y, Jiang X, Hao R, Wang Y, Chen Y, . . . Li X (2020). Effects of Oral Vitamin B1 and Mecobalamin on Dry Eye Disease. J Ophthalmol **2020**: 9539674.

Ren X, Chou Y, Wang Y, Jing D, Chen Y and Li X (2022). The Utility of Oral Vitamin B1 and Mecobalamin to Improve Corneal Nerves in Dry Eye Disease: An In Vivo Confocal Microscopy Study. NUTRIENTS **14**(18).

Robert P Y, Cochener B, Amrane M, Ismail D, Garrigue J S, Pisella P J, . . . Baudouin C (2016). Efficacy and safety of a cationic emulsion in the treatment of moderate to severe dry eye disease: a randomized controlled study. Eur J Ophthalmol **26**(6): 546-555.

Rodríguez Calvo-de-Mora M, Domínguez-Ruiz C, Barrero-Sojo F, Rodríguez-Moreno G, Antúnez Rodríguez C, Ponce Verdugo L, . . . Rocha-de-Lossada C (2022). Autologous versus allogeneic versus umbilical cord sera for the treatment of severe dry eye disease: a double-blind randomized clinical trial. Acta Ophthalmol **100**(2): e396-e408.

Rolando M, Villella E, Loreggian L, Marini S, Loretelli C, Fiorina P, . . . Barabino S (2023). Long-Term Activity and Safety of a Low-Dose Hydrocortisone Tear Substitute in Patients with Dry Eye Disease. Curr Eye Res **48**(9): 799-804.

Safarzadeh M, Azizzadeh P and Akbarshahi P (2017). Comparison of the clinical efficacy of preserved and preservative-free hydroxypropyl methylcellulose-dextran-containing eyedrops. J Optom **10**(4): 258-264.

Saffar Shahroodi A, Nejabat M, Nimrouzi M, Aghaei H, Salehi A and Rezaei Mokarram A (2019). Effects of intranasal administration of violet oil in dry eye disease. Clin Exp Optom **102**(6): 576-582.

Salim S, Kamath S J, Jeganathan S, Pai S G, Mendonca T M and Kamath A R (2023). Comparing the efficacy of sodium hyaluronate eye drops and carboxymethylcellulose eye drops in treating mild to moderate dry eye disease. Indian J Ophthalmol **71**(4): 1593-1597.

Sall K, Stevenson O D, Mundorf T K and Reis B L (2000). Two multicenter, randomized studies of the efficacy and safety of cyclosporine ophthalmic emulsion in moderate to severe dry eye disease. CsA Phase 3 Study Group. Ophthalmology **107**(4): 631-639.

Sánchez-González J-M, Silva-Viguera C, Sánchez-González M C, Capote-Puente R, De-Hita-Cantalejo C, Ballesteros-Sánchez A, . . . Gutiérrez-Sánchez E (2023). Tear Film Stabilization and Symptom Improvement in Dry Eye Disease: The Role of Hyaluronic Acid and Trehalose Eyedrops versus Carmellose Sodium. JOURNAL OF CLINICAL MEDICINE **12**(20): 6647.

Saubhagya Sindhu S D, Mirza Atif Beg, Sanjeev Kumar Mittal, Sushobhan Das Gupta (2015). Comparative evaluation of topical carboxymethyl cellulose either alone or in combination with topical corticosteroid in the treatment of dry eye in a tertiary-care teaching hospital. National Journal of Physiology, Pharmacy and Pharmacology **5**(3): 207-211.

Schechter B A (2006). Ketorolac during the induction phase of cyclosporin-A therapy. J Ocul Pharmacol Ther **22**(2): 150-154.

Schechter B A, Urbieta M, Bacharach J, Toyos M, Smyth-Medina R, Mitchell B, . . . Luchs J I (2022). Effect of OTX-101 in Patients with Dry Eye Disease at Day 14 of Treatment: Ocular Surface Endpoint Results from the Phase 2b/3 Clinical Trial. Clin Ophthalmol **16**: 4145-4151.

Schmidl D, Bata A M, Szegedi S, Aranha Dos Santos V, Stegmann H, Fondi K, . . . Garhöfer G (2020). Influence of Perfluorohexyloctane Eye Drops on Tear Film Thickness in Patients with Mild to Moderate Dry Eye Disease: A Randomized Controlled Clinical Trial. J Ocul Pharmacol Ther **36**(3): 154-161.

Schmidl D, Szalai L, Kiss O G, Schmetterer L and Garhöfer G (2021). A Phase II, Multicenter, Randomized, Placebo-Controlled, Double-Masked Trial of a Topical Estradiol Ophthalmic Formulation in Postmenopausal Women with Moderate-to-Severe Dry Eye Disease. Adv Ther **38**(4): 1975-1986.

Scuderi G, Contestabile M T, Gagliano C, Iacovello D, Scuderi L and Avitabile T (2012). Effects of phytoestrogen supplementation in postmenopausal women with dry eye syndrome: a randomized clinical trial. Can J Ophthalmol **47**(6): 489-492.

Semba C P, Torkildsen G L, Lonsdale J D, McLaurin E B, Geffin J A, Mundorf T K, . . . Ousler G W (2012). A phase 2 randomized, double-masked, placebo-controlled study of a novel integrin antagonist (SAR 1118) for the treatment of dry eye. Am J Ophthalmol **153**(6): 1050-1060.e1051.

Serrano-Morales J M, De-Hita-Cantalejo C, Sánchez-González M C, Bautista-Llamas M J and Sánchez-González J M (2022). Efficacy of 0.1% crosslinked hyaluronic acid, coenzyme Q10 and vitamin E in the management of dry eye disease in menopause patients receiving antidepressants. Eur J Ophthalmol **32**(1): 658-663.

Shah S, Badhu B P, Lavaju P, Chaudhary S and Sinha A K (2017). Efficacy of topical carboxymethyl cellulose 0.5% and cyclosporine A 0.05% in dry eye syndrome. Cogent Medicine **4**(1): 1321869.

Shemer A, Altarescu A, Nusbaum L, Vardi M, Dubinsky-Pertzov B, Hecht I, . . . Pras E (2023). Quantum Molecular Resonance Effects on Patients With Dry Eye Disease: A Randomized Controlled Trial. Cornea.

Sheppard J D, Torkildsen G L, Lonsdale J D, D'Ambrosio F A, Jr., McLaurin E B, Eiferman R A, . . . Semba C P (2014). Lifitegrast ophthalmic solution 5.0% for treatment of dry eye disease: results of the OPUS-1 phase 3 study. Ophthalmology **121**(2): 475-483.

Sheppard J D, Wirta D L, McLaurin E, Boehmer B E, Ciolino J B, Meides A S, . . . Krösser S (2021). A Water-free 0.1% Cyclosporine A Solution for Treatment of Dry Eye Disease: Results of the Randomized Phase 2B/3 ESSENCE Study. Cornea **40**(10): 1290-1297.

Shettle L, McLaurin E, Martel J, Seaman J W, 3rd and Weissgerber G (2022). Topical Anti-TNFα Agent Licaminlimab (OCS-02) Relieves Persistent Ocular Discomfort in Severe Dry Eye Disease: A Randomized Phase II Study. Clin Ophthalmol **16**: 2167-2177.

Shin J, Rho C R, Hyon J Y, Chung T Y, Yoon K C and Joo C K (2021). A Randomized, Placebo-Controlled Phase II Clinical Trial of 0.01% or 0.02% Cyclosporin A with 3% Trehalose in Patients with Dry Eye Disease. J Ocul Pharmacol Ther **37**(1): 4-11.

Shin M S, Kim J I, Lee M S, Kim K H, Choi J Y, Kang K W, . . . Kim T H (2010). Acupuncture for treating dry eye: a randomized placebo-controlled trial. Acta Ophthalmol **88**(8): e328-333.

Simmons P A, Carlisle-Wilcox C, Chen R, Liu H and Vehige J G (2015). Efficacy, safety, and acceptability of a lipid-based artificial tear formulation: a randomized, controlled, multicenter clinical trial. Clin Ther **37**(4): 858-868.

Simmons P A, Carlisle-Wilcox C and Vehige J G (2015). Comparison of novel lipid-based eye drops with aqueous eye drops for dry eye: a multicenter, randomized controlled trial. Clin Ophthalmol **9**: 657-664.

Simmons P A, Liu H, Carlisle-Wilcox C and Vehige J G (2015). Efficacy and safety of two new formulations of artificial tears in subjects with dry eye disease: a 3-month, multicenter, active-controlled, randomized trial. Clin Ophthalmol **9**: 665-675.

Singh K.K. (2022). Prospective, Randomized Comparative Assessment of Efficacy of Chloroquine Phosphate 0.03% And Sodium Carboxymethylcellulose 1% in Dry Eye. International Journal of Toxicological and Pharmacological Research **12**(4): 159-166.

Singla S, Sarkar L and Joshi M (2019). Comparison of topical cyclosporine alone and topical loteprednol with cyclosporine in moderate dry eye in Indian population: A prospective study. Taiwan J Ophthalmol **9**(3): 173-178.

Song Y, Yu S, He X, Yang L, Wu Y, Qin G, . . . Pazo E E (2022). Tear film interferometry assessment after intense pulsed light in dry eye disease: A randomized, single masked, sham-controlled study. Cont Lens Anterior Eye **45**(4): 101499.

Sosne G and Ousler G W (2015). Thymosin beta 4 ophthalmic solution for dry eye: a randomized, placebo-controlled, Phase II clinical trial conducted using the controlled adverse environment (CAE™) model. Clin Ophthalmol **9**: 877-884.

Stevenson D, Tauber J and Reis B L (2000). Efficacy and safety of cyclosporin A ophthalmic emulsion in the treatment of moderate-to-severe dry eye disease: a dose-ranging, randomized trial. The Cyclosporin A Phase 2 Study Group. Ophthalmology **107**(5): 967-974.

Su M Y, Perry H D, Barsam A, Perry A R, Donnenfeld E D, Wittpenn J R, . . . D'Aversa G (2011). The effect of decreasing the dosage of cyclosporine A 0.05% on dry eye disease after 1 year of twice-daily therapy. Cornea **30**(10): 1098-1104.

Supalaset S, Tananuvat N, Pongsatha S, Chaidaroon W and Ausayakhun S (2019). A Randomized Controlled Double-Masked Study of Transdermal Androgen in Dry Eye Patients Associated With Androgen Deficiency. Am J Ophthalmol **197**: 136-144.

Takamura E, Tsubota K, Watanabe H and Ohashi Y (2012). A randomised, double-masked comparison study of diquafosol versus sodium hyaluronate ophthalmic solutions in dry eye patients. Br J Ophthalmol **96**(10): 1310-1315.

Tan J, Jia T, Liao R and Stapleton F (2020). Effect of a formulated eye drop with Leptospermum spp honey on tear film properties. Br J Ophthalmol **104**(10): 1373-1377.

Tauber J, Davitt W F, Bokosky J E, Nichols K K, Yerxa B R, Schaberg A E, . . . Kellerman D J (2004). Double-masked, placebo-controlled safety and efficacy trial of diquafosol tetrasodium (INS365) ophthalmic solution for the treatment of dry eye. Cornea **23**(8): 784-792.

Tauber J, Evans D, Segal B, Li X Y, Shen W, Lu C, . . . Novack G D (2023). A phase 2a, double-masked, randomized, vehicle-controlled trial of VVN001 in subjects with dry eye disease. Ocul Surf **28**: 18-24.

Tauber J, Karpecki P, Latkany R, Luchs J, Martel J, Sall K, . . . Semba C P (2015). Lifitegrast Ophthalmic Solution 5.0% versus Placebo for Treatment of Dry Eye Disease: Results of the Randomized Phase III OPUS-2 Study. Ophthalmology **122**(12): 2423-2431.

Tauber J, Schechter B A, Bacharach J, Toyos M M, Smyth-Medina R, Weiss S L, . . . Luchs J I (2018). A Phase II/III, randomized, double-masked, vehicle-controlled, dose-ranging study of the safety and efficacy of OTX-101 in the treatment of dry eye disease. Clin Ophthalmol **12**: 1921-1929.

Taylor M, Ousler G, Torkildsen G, Walshe C, Fyfe M C T, Rowley A, . . . Duggal A (2019). A phase 2 randomized, double-masked, placebo-controlled study of novel nonsystemic kinase inhibitor TOP1630 for the treatment of dry eye disease. Clin Ophthalmol **13**: 261-275.

Titiyal J S, Kaur M, Falera R, Bharghava A, Sah R and Sen S (2019). Efficacy and Safety of Topical Chloroquine in Mild to Moderate Dry Eye Disease. Curr Eye Res **44**(12): 1306-1312.

Tong L, Htoon H M, Hou A, Acharya R U, Tan J H, Wei Q P, . . . Lim P (2018). Acupuncture and herbal formulation compared with artificial tears alone: evaluation of dry eye symptoms and associated tests in randomised clinical trial. BMJ Open Ophthalmol **3**(1): e000150.

Torkildsen G L, Pattar G R, Jerkins G, Striffler K and Nau J (2022). Efficacy and Safety of Single-dose OC-02 (Simpinicline Solution) Nasal Spray on Signs and Symptoms of Dry Eye Disease: The PEARL Phase II Randomized Trial. Clin Ther **44**(9): 1178-1186.

Toshida H, Funaki T, Ono K, Tabuchi N, Watanabe S, Seki T, . . . Murakami A (2017). Efficacy and safety of retinol palmitate ophthalmic solution in the treatment of dry eye: a Japanese Phase II clinical trial. Drug Des Devel Ther **11**: 1871-1879.

Toshida H, Funaki T, Ono K, Tabuchi N, Watanabe S, Seki T, . . . Murakami A (2021). Erratum: Efficacy and Safety of Retinol Palmitate Ophthalmic Solution in the Treatment of Dry Eye: A Japanese Phase II Clinical Trial [Corrigendum]. Drug Des Devel Ther **15**: 813-816.

Utsunomiya T, Kawahara A, Hanada K and Yoshida A (2017). Effects of Diquafosol Ophthalmic Solution on Quality of Life in Dry Eye Assessed Using the Dry Eye-Related Quality-of-Life Score Questionnaire: Effectiveness in Patients While Reading and Using Visual Display Terminals. Cornea **36**(8): 908-914.

van Setten G B, Baudouin C, Horwath-Winter J, Böhringer D, Stachs O, Toker E, . . . Müller-Lierheim W G K (2020). The HYLAN M Study: Efficacy of 0.15% High Molecular Weight Hyaluronan Fluid in the Treatment of Severe Dry Eye Disease in a Multicenter Randomized Trial. J Clin Med **9**(11).

Villani E, Laganovska G, Viola F, Pirondini C, Baumane K, Radecka L, . . . Ratiglia R (2011). A multicenter, double-blind, parallel group, placebo-controlled clinical study to examine the safety and efficacy of T-Clair SPHP700-3 in the management of mild to moderate dry eye in adults. Cornea **30**(3): 265-268.

Vogel R, Crockett R S, Oden N, Laliberte T W and Molina L (2010). Demonstration of efficacy in the treatment of dry eye disease with 0.18% sodium hyaluronate ophthalmic solution (vismed, rejena). Am J Ophthalmol **149**(4): 594-601.

Wang I J, Lin I C, Hou Y C and Hu F R (2007). A comparison of the effect of carbomer-, cellulose- and mineral oil-based artificial tear formulations. Eur J Ophthalmol **17**(2): 151-159.

Watts P, Sahai A, Kumar P R, Shamshad M A, Trivedi G K and Tyagi L (2020). A prospective study to assess the role of vitamin D individually and in combination with cyclosporine in the treatment of dry eye in patients with deficient serum 25(OH)D levels. Indian J Ophthalmol **68**(6): 1020-1026.

Wirta D, Torkildsen G L, Boehmer B, Hollander D A, Bendert E, Zeng L, . . . Nau J (2022). ONSET-1 Phase 2b Randomized Trial to Evaluate the Safety and Efficacy of OC-01 (Varenicline Solution) Nasal Spray on Signs and Symptoms of Dry Eye Disease. Cornea **41**(10): 1207-1216.

Wirta D, Vollmer P, Paauw J, Chiu K H, Henry E, Striffler K, . . . Nau J (2022). Efficacy and Safety of OC-01 (Varenicline Solution) Nasal Spray on Signs and Symptoms of Dry Eye Disease: The ONSET-2 Phase 3 Randomized Trial. Ophthalmology **129**(4): 379-387.

Wirta D L, Senchyna M, Lewis A E, Evans D G, McLaurin E B, Ousler G W, . . . Hollander D A (2022). A randomized, vehicle-controlled, Phase 2b study of two concentrations of the TRPM8 receptor agonist AR-15512 in the treatment of dry eye disease (COMET-1). The Ocular Surface **26**: 166-173.

Wirta D L, Torkildsen G L, Moreira H R, Lonsdale J D, Ciolino J B, Jentsch G, . . . Krösser S (2019). A Clinical Phase II Study to Assess Efficacy, Safety, and Tolerability of Waterfree Cyclosporine Formulation for Treatment of Dry Eye Disease. Ophthalmology **126**(6): 792-800.

Wu Y, Jin X, Mou Y, Yuan K, Min J and Huang X (2021). A 4-week, randomized, double-masked study to evaluate efficacy of deproteinized calf blood extract eye drops versus sodium hyaluronate 0.3% eye drops in dry eye patients with ocular pain. Ann Palliat Med **10**(4): 3617-3625.

Yang G, Kong X, Guo X, Yang Y, Xie C, Lu Y, . . . Ma X (2023). Effects of electroacupuncture on dry eye: A pilot randomized controlled trial. Acta Ophthalmol **101**(3): e315-e326.

Yang J M, Choi W, Kim N and Yoon K C (2015). Comparison of Topical Cyclosporine and Diquafosol Treatment in Dry Eye. Optom Vis Sci **92**(9): e296-302.

Yang G, Wang Y M (2021). Clinical Efficacy of Sodium Hyaluronate Eye Drops Combined With Pranoprofen in the Treatment of Patients with Dry Eye. Indian Journal of Pharmaceutical Sciences **83**(5): spl issue "1-5".

Zheng N and Zhu S Q (2023). Randomized controlled trial on the efficacy and safety of autologous serum eye drops in dry eye syndrome. World J Clin Cases **11**(28): 6774-6781.
